# LncGBP9/miR-34a axis drives macrophages toward a phenotype conducive for spinal cord injury repair via STAT1/STAT6 and SOCS3

**DOI:** 10.1186/s12974-020-01805-5

**Published:** 2020-04-28

**Authors:** Jiahui Zhou, Zhiyue Li, Tianding Wu, Qun Zhao, Qiancheng Zhao, Yong Cao

**Affiliations:** 1 Department of Orthopedics, The Third Xiangya Hospital, Central South University, Changsha, 410013 China; 2 Department of Spine Surgery, Xiangya Hospital of Central South University, Changsha, 410008 PR of China

**Keywords:** spinal cord injury (SCI), Macrophage polarization, lncGBP9, SOCS3, STAT1/STAT6, MiR-34a

## Abstract

**Background:**

Acute spinal cord injury (SCI) could cause mainly two types of pathological sequelae, the primary mechanical injury, and the secondary injury. The macrophage in SCI are skewed toward the M1 phenotype that might cause the failure to post-SCI repair.

**Methods:**

SCI model was established in Balb/c mice, and the changes in macrophage phenotypes after SCI were monitored. Bioinformatic analyses were performed to select factors that might regulate macrophage polarization after SCI. Mouse bone marrow-derived macrophages (BMDMs) were isolated, identified, and induced for M1 or M2 polarization; the effects of lncRNA guanylate binding protein-9 (lncGBP9) and suppressor of cytokine signaling 3 (SOCS3) on macrophages polarization were examined in vitro and in vivo. The predicted miR-34a binding to lncGBP9 and SOCS3 was validated; the dynamic effects of lncGBP9 and miR-34a on SOCS3, signal transducer and activator of transcription 1 (STAT1)/STAT6 signaling, and macrophage polarization were examined. Finally, we investigated whether STAT6 could bind the miR-34a promoter to activate its transcription.

**Results:**

In SCI Balb/c mice, macrophage skewing toward M1 phenotypes was observed after SCI. In M1 macrophages, lncGBP9 silencing significantly decreased p-STAT1 and SOCS3 expression and protein levels, as well as the production of Interleukin (IL)-6 and IL-12; in M2 macrophages, lncGBP9 overexpression increased SOCS3 mRNA expression and protein levels while suppressed p-STAT6 levels and the production of IL-10 and transforming growth factor-beta 1 (TGF-β1), indicating that lncGBP9 overexpression promotes the M1 polarization of macrophages. In lncGBP9-silenced SCI mice, the M2 polarization was promoted on day 28 after the operation, further indicating that lncGBP9 silencing revised the predominance of M1 phenotype at the late stage of secondary injury after SCI, therefore improving the repair after SCI. IncGBP9 competed with SOCS3 for miR-34a binding to counteract miR-34a-mediated suppression on SOCS3 and then modulated STAT1/STAT6 signaling and the polarization of macrophages. STAT6 bound the promoter of miR-34a to activate its transcription.

**Conclusions:**

In macrophages, lncGBP9 sponges miR-34a to rescue SOCS3 expression, therefore modulating macrophage polarization through STAT1/STAT6 signaling. STAT6 bound the promoter of miR-34a to activate its transcription, thus forming two different regulatory loops to modulate the phenotype of macrophages after SCI.

## Background

Spinal cord injury (SCI) is one of the most critical global contributors to disability and related death; however, the treatment efficacy of SCI is still unsatisfactory [1–3]. Acute SCI could cause mainly two types of pathological sequelae, the primary mechanical injury and the secondary injury [4]. Direct mechanical trauma could cause the primary SCI, followed by the secondary injury through activating several pathophysiological processes, such as inflammation, dysregulation of microvascular perfusion, deregulated generation of free radicals, dysregulation of cell apoptosis, and broken ionic homeostasis [5–9], which would directly lead to the destruction of intact axonal tracts and hinder the structural and functional recoveries after initial SCI.

During both the acute and chronic phases of the secondary injury when the central nervous system (CNS) evokes innate and adaptive immunity [9, 10], inflammatory responses have been regarded as the primary issue. Recent studies have shown that the sequential activation of immune cells, including resident and recruited subtypes, may play an important role in the secondary inflammatory/immune responses after CNS injury, including SCI. The macrophage is a critical cell type in the innate immune response within CNS. In CNS injury, macrophages are heterogeneous and comprised predominantly of two groups: specialized CNS-resident macrophages (microglia) and bone marrow-derived macrophages (BMDMs). Microglial cells are renewed by local proliferation, arrive in the CNS from yolk sacks in development, and are responsible for surveying the CNS parenchyma and aid in synaptic pruning [11–13]. BMDMs are often localized mainly in the margins of the lesion site following SCI, while the microglia cells are usually distributed in the lesion core and its margins. After injury, infiltrating BMDMs migrate to the epicenter of injury, while microglia-derived macrophages localize to the edges of the lesion [12]. In other words, the majority of macrophages in the lesion site are BMDMs rather than locally activated microglia. These two populations of macrophages with different locations have different functions. Residential microglia-derived macrophages form a border that seems to seal the lesion and block the spread of damage, whereas BMDMs enter the epicenter of the injured spinal cord and phagocytize apoptotic and necrotic cells and clear tissue debris such as myelin debris [14].

Macrophages are phenotypically dynamic in both morphology and function, ranging from resting ramified steady-state (M0) to a pro-inflammatory (M1) or an anti-inflammatory phenotype (M2) [15]. In fact, the situation might be more complicated in vivo. After SCI, the collective actions of the non-specific and adaptive immune system can be recruited and serve various functions, which are both neurotoxic and neuroprotective. The interferon-γ (IFN-γ) and prototypical T-helper 1 cytokine (TH1) can activate and induce macrophages to produce cytotoxic mediators (reactive oxygen and nitrogen species) and pro-inflammatory cytokines, IFN-γ, tumor necrosis factor-alpha (TNF-α), C-C motif chemokine ligand 5 (CCL5), IL-23, IL-12, IL-6, and IL-1β, and increase their ability to kill pathogens within cells. By contrast, the IL-13, IL-4, TH2, and so on inhibit macrophages from producing pro-inflammation cytokines [16, 17], and increase their ability to kill extracellular pathogens such as parasite infection [18, 19]. These two different macrophage phenotypes induced by either TH1 (IFN-γ and TLR signaling) or TH2 (IL-13 and IL-4) are known as M1 or “classically activated” macrophage, while the latter is called M2 or “alternatively activated” macrophage [20]. Within a few hours after SCI, macrophages first polarized into M1 macrophages in response to IFN-γ, lipopolysaccharides (LPS), TNF-α, and other stimuli, and reaches a peak on day 1 after SCI. Macrophage surface Toll-like receptors (TLRs) are activated and then induce the recruitment of downstream protein myeloid differentiation factor 88 (MyD88), the activation of the downstream pathways, including nuclear factor kappa B (NF-κB), Janus kinase (JAK)-signal transducer and activator of transcription (STAT), c-Jun N-terminal kinases (JNK), mitogen-activated protein kinase (MAPK), and phosphoinositide 3-kinases (PI3K)/protein kinase B (Akt or PKB) [21], and promote the polarized macrophages to release TNF-α, IL-β, IL-6, other inflammatory factors [22] and chemokines (CCL8 and CCL9), and cyclooxygenase (Cox), which in turn promote the differentiation of more macrophages to M1. Later, the polarized M1 macrophages exhibit stronger phagocytosis and antigen-presenting ability. Moreover, a large number of M1 macrophage-secreted inflammatory cytokines, reactive oxygen species (ROS), reactive nitrogen (RNS), prostaglandin (PGE2), and other active substances cause damage to neurons and glia, leading to neuronal apoptosis [23]. As for M2 macrophages, cell surface receptors bind IL-4 and IL-13 to promote signal transducer and activator of transcription 6 (STAT6) phosphorylation, therefore stimulating the macrophage polarization into M2 type [24]. The markers of M2 macrophages include arginase (Arg-1), resistin-like molecules (Fizz-1), IL-10, TGF-β, mannitol receptors (including CD163, CD204, CD206), etc. [25]. M2 macrophages highly express IL-10, IL-4, IL-13, TGF-β, and neurotrophic factor, which can inhibit neuronal apoptosis and the proinflammatory effects of M1 macrophages, promoting nerve tissue repair. There are three subtypes of M2 macrophages including M2a/b/c; M2a macrophages appear on days 1~3 after SCI, highly express CD206 and Arg1, and exert anti-inflammatory and repair functions [26]; M2b appears on days 3~7 after SCI, express high IL-10 and low Arg1; M2c appears the latest and could inhibit the production of inflammatory cytokines and inflammation [27].

Macrophages are widely malleable in functions, allowing them to convert from one phenotype to another under the broad stimuli in the post-SCI inflammatory microenvironment. M1 macrophages play a detrimental role after SCI, while M2 macrophages play a promotive role in the regenerative growth responses in adult sensory axons. After SCI, increased macrophages skewing toward the M1 phenotype and the decreased number of M2 macrophages may lead to or even aggravate the secondary injury. Previously, it has been reported that the angiotensin-converting enzyme (ACE)-C domain overexpression in macrophages would result in the transformation of macrophages toward the M1 phenotype in tumor microenvironment, accompanied by enhanced activation of NF-κB and signal transducer and activator of transcription 1 (STAT1) and attenuated activation of STAT3/6 [28]. Yao et al. reported that the M1 polarization could be hindered by a crucial immune inhibitory receptor, namely programmed cell death 1 (PD-1), via suppressing the phosphorylation of STAT1; in the meantime, PD-1 also promoted the M2 polarization via enhancing the phosphorylation of STAT6 [29]. Consistently, the M1 polarization was enhanced by activating STAT1 and NF-κB in PD-1 knockout mice [29]. Therefore, investigating the factors and mechanisms of regulating STAT1/STAT6 signaling may help to understand the mechanism of the M1/2 macrophage phenotype switch.

During the past decades, non-protein coding RNAs (ncRNAs) have been regarded as key regulators by playing diverse roles in not only fundamental biological but also pathological processes [30, 31]. Among them, microRNA (miRNA) and long noncoding RNAs (lncRNAs) are the most well-known. miRNAs induce either mRNA degradation or block mRNA translation depending on the complete or incomplete complementarity [32], while lncRNAs could serve as competing endogenous RNAs (ceRNAs) to counteract miRNA-mediated inhibition on miRNA downstream transcripts, therefore exerting their biological functions [33–35]. Like miRNA, there is new evidence that lncRNAs might be a novel type of regulator macrophage immune response [36]. LncRNAs have been reported to be partially responsible for the gene expression dysregulation during macrophage polarization [37]. Huang et al. [38] also identified differentially expressed lncRNAs in M1- or M2-polarized macrophages. Of these deregulated lncRNAs, lncRNA TCONS_00019715 has higher expression in M1 macrophages than that in M2 macrophages. When proinflammatory macrophages convert to anti-inflammatory macrophages, TCONS_00019715 expression decreases. However, it increases when anti-inflammatory macrophages convert to proinflammatory phenotype. Knockdown of TCONS_00019715 diminishes the expression of proinflammatory macrophage markers and increases the expression of anti-inflammatory markers. TCONS_00019715 promotes macrophage transition to proinflammatory macrophages by downregulating P21 (RAC1)-activated kinase 1 (PAK1), an important regulator of cytoskeletal remodeling and cell motility in mononuclear phagocytic system [38]. Another lncRNA, lncRNA E330013P06, was found to regulate proinflammatory gene expression and foam cell formation in macrophages [39]. Based on these previous findings, we hypothesize that lncRNAs may participate in macrophage polarization by regulating related molecules and STAT1/STAT6 signaling pathways, most possibly in a miRNA-dependent manner.

In the present study, we conducted the SCI model in Balb/c mice and examined the expression of M1/2 macrophage markers on days 1, 3, 7, 14, and 28 after the operation to monitor the changes in the macrophage phenotypes. Next, by downloading and analyzing online microarray profiles reporting differentially expressed lncRNAs and genes in M1/2 macrophages, we selected lncRNAs and genes related to macrophage polarization, namely lncGBP9 in mice and suppressor of cytokine signaling 3 (SOCS3). Mouse BMDMs were isolated, identified, induced to differentiate into M1/2 macrophages, and examined for expression of SOCS3, STAT1, and STAT6 and cytokine production. Next, the effects of lncGBP9 on macrophage polarization, SOCS3 expression, and STAT1/STAT6 signaling were evaluated in vitro and in vivo. Since miR-34a has been reported to promote the M2 macrophage polarization [40] and be predicted to target lncGBP9 and SOCS3, we further investigated whether lncGBP9 could compete with SOCS3 for miR-34a binding, thereby counteracting miR-34a-mediated SOCS3 suppression. The predicted bindings of miR-34a to lncGBP9 and SOCS3 were validated and the dynamic effects of lncGBP9 and miR-34a on SOCS3, STAT1/STAT6 signaling, and macrophage polarization were examined. Finally, we investigated whether STAT6 could bind the miR-34a promoter to activate its transcription. In summary, we provide a novel mechanism by which the lncGBP9/miR-34a axis modulates STAT1/STAT6 to affect macrophage polarization via SOCS3.

## Methods

### Spinal cord injury model in Balb/c mice

Balb/c mice (The SLAC experimental animal center, Shanghai, China) received a moderate midthoracic (T9–10) spinal cord injury (SCI), as described previously [41]. Sham mice received a laminectomy without SCI. To identify the SCI model in mice, the study collected tissue in lesion epicenter on 1, 3, 7, 14, or 28 days after SCI and examined for the M1 macrophage marker CD16/32 and M2 macrophage marker Arg1 by immunofluorescence (IF) staining; the mRNA expression of M1 macrophage markers iNOS, CD16/32, and IFN-γ; and M2 macrophage markers Arg1, CD206, and IL-4 by real-time PCR and Immunoblotting. Locomotor recovery of mice was assessed by two persons using the Basso Mouse Scale (BMS) [42] open field test at 1, 3, 7, 14, and 28 days after injury following the methods described previously [42, 43]. All procedures involving animals were approved by the Central South University Research Ethics Committee.

### Isolation and identification of mouse bone marrow-derived macrophages

BMDMs were isolated from the bilateral femurs and tibias of adult Balb/c mice and then cultured as described previously [44, 45]. Twenty percent supernatant from L929 cells was added to stimulate BMDMs to differentiate into macrophages for 7 days [46].

### Induction and identification of M0 macrophages polarized toward M1/2 macrophages

The induction of M0 macrophages differentiation into M1 or M2 macrophages was conducted with 100 ng/ml LPS (Sigma-Aldrich) + 20 ng/ml IFN-γ (eBioscience) or 20 ng/ml IL-4 (eBioscience), respectively, for 48 h following the methods described previously [44–46]. Then, cells were harvested for ELISA, real-time PCR, and immunoblotting assays.

### Recombinant adenoviruses preparation, transduction, and injection

The recombinant adenoviruses expressing lncGBP9 shRNA or lncGBP9-overexpressing fragment or scramble RNA (NC shRNA) were generated using the AdEasyTM Vector System (Invitrogen) following the methods described before [47]. For in vitro experiments, adv-sh-NC, adv-sh-lncGBP9, adv-NC, and adv-lncGBP9 were then diluted in PBS and administered at a concentration of 2 × 10^7^ pfu/well in 12-well plate. Twenty-four hours later, M0 macrophage were stimulated to polarization followed the methods mentioned above. Then, cells were harvested for ELISA, real-time PCR, and immunoblotting assays. For in vivo experiments, total 1 μl of adv-sh-NC or adv-sh-lncGBP9 (1 × 10^10^ pfu/ml, *n* = 5) was injected to the injured spinal cord at a depth of 0.5 mm and 1 mm (each depth 0.5 μl) using 5-μl Hamilton syringe, each injection was performed at 0.2 μl/min. Before withdrawing the syringe, the needle was left in place for a further 2 min to avoid the viral leakage. The dorsal muscle and skin were then sutured. Twenty-eight days later, the mice were sacrificed for further experiments.

### Immunofluorescence staining

Spine cord tissues were fixed in 4% paraformaldehyde and dehydrated using 30% sucrose overnight. After embedding into OCT compound (Tissue Tek), tissues were cut into 16 μm section. Sections were blocked using 5% normal goat serum and then were incubated with the diluted primary antibody specific to F4/80 (ab111101, Abcam, MA, USA), CD16/32 (Catalog # 14-0161-82, Invitrogen, Waltham, MA, USA), and Arg1 (sc-271430, Santa Cruz, USA), overnight at 4 °C. Cy3 or FITC-conjugated secondary antibody (Santa Cruz) were incubated with sections at room temperature for 1 h. For cellular immunofluorescence, cells were fixed with 4% paraformaldehyde and incubated with the primary antibody specific to F4/80 and CD11b (ab8878) and then incubated with Cy3 or FITC-conjugated secondary antibodies. DAPI (C1002, Beyotime, China) was used to stain the nucleus in tissue sections and cells before capturing images. The images were acquired using a fluorescence microscope (Nikon, Japan).

### Immunoblotting

Immunoblotting was performed as previously described [48] using the following primary antibodies: anti-iNOS (ab15323, Abcam), anti-CD16 (ab203883), anti-IFNγ (500-P119, PeproTech, Rocky Hill, NJ, USA), anti-Arg1 (sc-271430, Santa Cruz), anti-CD206 (ab64693, Abcam), anti-IL-4 (ab11524, Abcam), anti-STAT1 (ab3987, Abcam), anti-p-STAT1 (ab30645, Abcam), anti-STAT6 (ab32520, Abcam), anti-p-STAT6 (ab28829, Abcam), anti-SOCS3 (ab16030, Abcam), and the HRP-conjugated secondary antibody (1:5000). The plots were visualized by ECL Plus (Thermo).

### Real-time PCR

Total RNA was extracted from target cells by using Trizol reagent (Invitrogen) and the expression of miRNA or mRNA was examined following the methods previously described [49] using a Hairpin-it TM miRNAs qPCR kit (Genepharma, Shanghai, China) or an SYBR Green PCR Master Mix (QIAGEN), respectively. U6 or β-actin expression was used as an endogenous normalization, respectively. The threshold cycle (Ct) was determined, and relative mRNA and miRNA levels were calculated using 2^-ΔΔCt^ methods. The primer sequences were listed in Table S1.

### ELISA

Cell culture medium was collected for ELISA assay using human IL-6, IL-12, IL-10, and TGFβ1 ELISA kits according to the manufacturer’s instructions (Santa Cruz Biotechnology, Santa Cruz, CA, USA) following the methods described previously [50]. The specific binding optical density was assayed immediately at 450 nm with a spectrophotometer (Bio-Rad Laboratories).

### Cell transfection

Commercial SOCS3 overexpression vector, STAT6 overexpression vector, small interference RNA (si) for SOCS3 and STAT6, and miR-34a mimics and inhibitor were obtained from RiboBio, Guangzhou, China. All transfections were performed with the help of Lipo2000 (Invitrogen) according to the manufacture’s instruction. Twenty-four hours after transfection, the M0 macrophages were polarized to M1/2 macrophages. Then, 48 h later, cells were harvested for ELISA, real-time PCR, and immunoblotting assays.

### Luciferase reporter assays

For the validation of the binding of miR-34a to GBP9 or SOCS3 3′-UTR, the wild-type GBP9 or SOCS3 3′-UTR luciferase reporter vector was constructed by cloning the fragment of GBP9 or SOCS3 3′-UTR to the Renilla psiCHECK2 vector (Promega, Madison, WI, USA) and named wt-GBP9/wt-SOCS3 3′-UTR; the mutant-type GBP9 or SOCS3 3′-UTR vector was constructed by mutating the predicted miR-34a binding site in GBP9 or SOCS3 3′-UTR and named mut-GBP9/mut-SOCS3 3′-UTR. These vectors were co-transfected in HEK293 cells with miR-34a mimics/inhibitor. For validation of the binding of STAT6 to the promoter of miR-34a, wild- and mutant-type miR-34a promoter luciferase reporter vectors were constructed; STAT6 and wt- or mut-miR-34a promoter were then co-transfected in M0 macrophages followed by M2 polarization; the luciferase activity was detected using the Dual-Luciferase Reporter Assay System (Promega). The primers used for luciferase reporter vector construction were listed in Table S1.

### RNA Immunoprecipitation

NC mimics, miR-34a mimics, adv-NC, or adv-lncGBP9 was transfected or transduced in M0 macrophages for 24 h. Then, cells were under M1 polarization. Forty-eight hours later, cells were harvested for RNA immunoprecipitation (RIP) assays. RIP analysis was performed on M1 macrophages using a Magna RIP RNA-Binding Protein Immunoprecipitation Kit (17-700, Millipore, Burlington, MA, USA) following the methods described previously [51]. The levels of GBP9, SOCS3, and miR-34a in the protein argonaute-2 (AGO2) or IgG immunoprecipitates were measured by real-time PCR using SYBR Green PCR mix (QIAGEN). IgG was used as a negative control. The primers were listed in the Table S1.

### Chromatin immunoprecipitation

STAT6 overexpression vector or NC vector was transfected in M0 macrophages for 24 h. Then, cells were under M2 polarization. Forty-eight hours later, cells were harvested for chromatin immunoprecipitation (ChIP) assays. ChIP assays were performed following the methods described previously [52] using antibodies against STAT6 (ab32520, Abcam), a positive control antibody (RNA polymerase II), and a negative control non-immune IgG. The immunoprecipitated DNA was cleaned, released, eluted, and used for real-time PCR. The fold-enrichment (FE) was calculated as previously described [52].

### Statistical analyses

All the data of results from at least three independent experiments in the present study are first processed by SPSS17.0 (IBM, Armonk, NY, USA) and presented as the mean ± S.D *n* ≥ 3 independent experiments. The statistical comparison between means was conducted using a Student *t* test where applicable. Differences among more than two groups were analyzed using one-way ANOVA. *P < 0.05; **P < 0.01.

## Results

### Alteration of macrophage phenotype during SCI

**Fig. 1:**
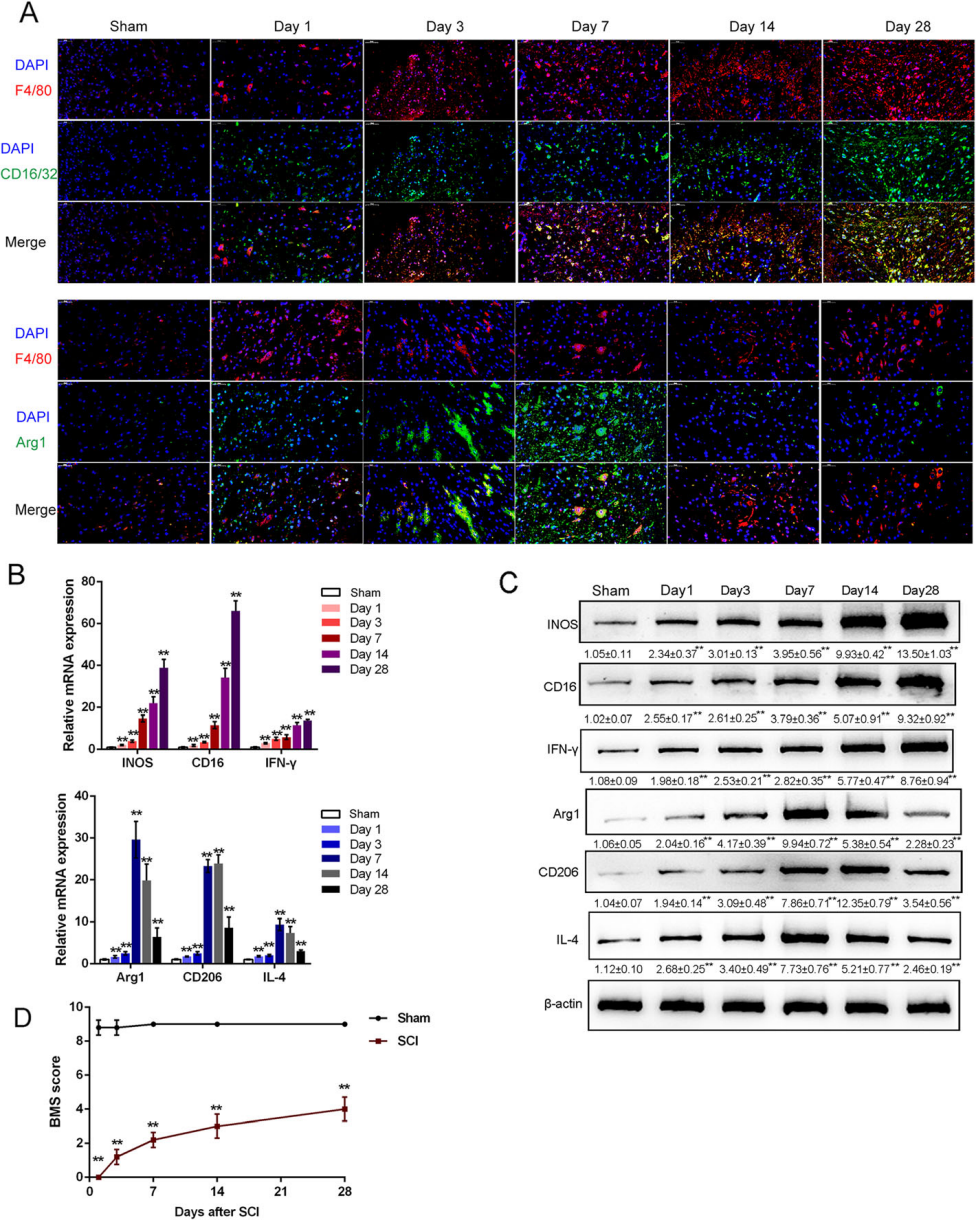
Alternation of macrophage phenotype during spinal cord injury (SCI). **a** SCI model was constructed in Balb/c mice as described in M&M section. *n* = 5 in each group. The content and distribution of M1 macrophage marker CD16/32 and M2 macrophage marker Arg1 in spinal cords of sham mice (28 day after sham operation) and SCI mice were examined by Immunofluorescence (IF) staining on days 1, 3, 7, 14, and 28 after treatment. **b** The mRNA expression and **c** the protein levels of M1 macrophage markers CD16, iNOS, and IFN-γ and M2 macrophage markers Arg1, CD206, and IL-4 were examined by real-time PCR (*n* = 5) and immunoblotting in sham group (28 days after sham operation) or SCI group days 1, 3, 7, 14, and 28 after treatment. *n* = 3. **d** The BMS scores in sham group or SCI group days 1, 3, 7, 14, and 28 after treatment were determined. (*n* = 5). *P < 0.05, **P < 0.01

To investigate the macrophage phenotypes during SCI, we established a mouse model of SCI using Balb/c mice following the methods described previously [41]. To identify the SCI model, we measured the BMS scores of the content and distribution of macrophage marker F4/80, M1 macrophage marker CD16/32, and M2 macrophage marker Arg1 in spinal cords from the sham group and SCI mouse on days 1, 3, 7, 14, and 28 after the operation. As revealed by IF staining, compared to the sham group, the fluorescence intensity representing F4/80 (red) and CD16/32 (green on the upper panel) gradually increased in a time-dependent manner in SCI group (Fig. 1a); however, the fluorescence intensity representing Arg1 (green on the lower panel) increased moderately on days 1 and 3, reached a sharp peak value on day 7, and then gradually decreased on day 14 and day 28 (Fig. 1a). Consistently, the mRNA expression and the protein levels of M1 macrophage markers CD16, iNOS, and IFN-γ and M2 macrophage markers Arg1, CD206, and IL-4 emerged similar trend. M1 macrophage markers increased after SCI in a time-dependent manner from day 1 to day 28, while M2 macrophage markers increased moderately on days 1 and 3, reached sharp peak values on day 7 or 14, and then decreased gradually (Fig. 1b, c). The BMS score results confirmed that the SCI model was successfully established (Fig. 1d). These data indicate that the macrophages in the spinal cord are skewed toward M1 after SCI that might participate in the dysfunction of SCI repair.

### Selection of lncRNAs and genes related to macrophage polarization

As we have mentioned, the deregulation and dysfunction of lncRNAs in the process of a pro-inflammatory (M1) to an anti-inflammatory phenotype (M2) have been observed [38, 53]. In the present study, we attempted to identify lncRNAs related to the skewing macrophage toward the M1 phenotype after SCI. The study downloaded and analyzed microarray profiles (GSE117040 and GSE5099), which reported upregulated lncRNAs in M1 macrophages. A total of four lncRNAs were reported to be upregulated in M1 macrophages by both profiles; there are homologous genes for lncRNA GBP1P1 (GBP9 in mice) and LINC00869 (Fam91a1 in mice) in mice (Fig. S1A).

**Fig. 2:**
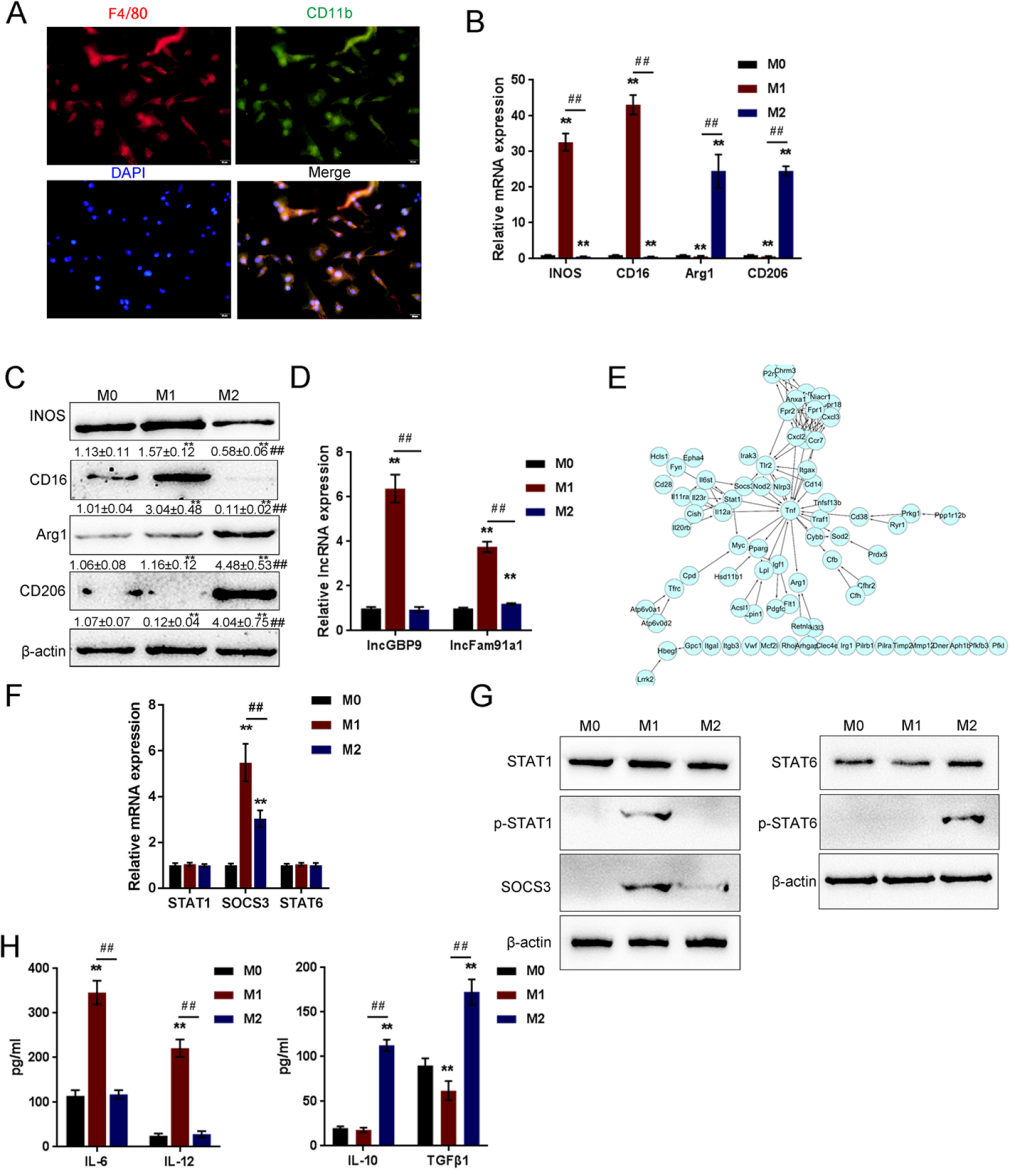
Selection of lncRNAs and genes related to macrophage polarization. **a** Mouse bone marrow-derived macrophages (BMDMs) were isolated and identified by examining macrophage markers F4/80 and CD11b using IF staining. **b** BMDMs were induced for differentiating towards M1 or M2 macrophages; the mRNA expression of M1 macrophage markers iNOS and CD16 and M2 macrophage markers Arg1 and CD206 were examined in M0, M1, and M2 macrophages by real-time PCR (*n* = 5). **c** The protein levels of M1 macrophage markers iNOS and CD16 and M2 macrophage markers Arg1 and CD206 were examined by Immunoblotting (*n* = 3). **d** The expression of GBP9 and Fam91a1 were examined by real-time PCR (*n* = 5). **e** STRING analyses on differentially-expressed genes reported previously. Tnf, Socs3, and Stat1 are key factors in macrophage polarization. **f**–**g** The mRNA expression and protein levels of STAT1, p-STAT1, SOCS3, STAT6, and p-STAT6 in M0, M1, and M2 macrophages determined by real-time PCR (*n* = 5) and immunoblotting (*n* = 3). **h** The production of cytokines, including IL-6, IL-12, IL-10, and TGF-β1 in M0, M1, and M2 macrophages determined by ELISA (*n* = 3). *P < 0.05, **P < 0.01, compared to control group; #*P* < 0.05, ##*P* < 0.01, compared to M1 group

Next, to further validate the involvement of these lncRNAs, we isolated BMDMs and identified them by examining macrophage markers F4/80 and CD11b using IF staining (Fig. 2a). BMDMs (M0 macrophages) were then induced for differentiating toward M1 or M2 macrophages; the mRNA expression and protein levels of M1 macrophage markers iNOS and CD16 and M2 macrophage markers Arg1 and CD206 were examined to identify different subtypes. As shown in Fig. 2b, c, iNOS and CD16 were significantly upregulated in M1 subtype while downregulated in M2 subtype; Arg1 and CD206 were remarkably upregulated in M2 subtype while downregulated in M1 subtype, indicating the successful induction. As revealed by real-time PCR, the expression of GBP9 and Fam91a1 were both significantly upregulated in M1 macrophages, GBP9 more upregulated (Fig. 2d), indicating that GBP9 might be involved in macrophage M1/2 polarization. Reportedly, GBP1P1 is a pseudogene of the guanylate-binding protein of guanylate-binding protein (GBP); this family is also involved in macrophage functions, such as IFN-γ-mediated macrophage activation and immune defense [54]. More importantly, based on microarray profile or RNA-seq analyses GSE5099 (Fig. S1B), GSE117040 (Fig. S1C), E-GEOD-57494 (Fig. S1D), E-MTAB-2399 (Fig. S1E), and GSE40885 (Fig. S1F), lncRNA GBP1P1 is specifically highly expressed in human M1 macrophages and could be rapidly upregulated after treatment with M1-inducing factors LPS and IFN-γ. Thus, GBP1P1 (lncGBP9 in mice) was selected for further experiments.

We then performed search tool for the retrieval of interacting genes/proteins (STRING) analyses on differentially expressed genes in M1 macrophages reported previously [38, 55] to identify key regulators of the switch from a pro-inflammatory (M1) to an anti-inflammatory phenotype (M2). As revealed by STRING analyses, Tnf, SOCS3, and STAT1 are key factors in macrophage polarization (Fig. 2e). Next, the mRNA expression and protein levels of M1-related STAT1 and p-STAT1, M2-related STAT6 and p-STAT6, and SOCS3 were examined in M0, M1, and M2 macrophages. As shown in Fig. 2f, g, p-STAT1 protein levels and SOCS3 mRNA and protein levels were dramatically upregulated in M1 macrophages while p-STAT6 was upregulated in M2 macrophages. In the meantime, the production of STAT1 downstream cytokines, including IL-6 and IL-12, was increased in M1 macrophages while that of STAT6 downstream IL-10 and transforming growth factor-beta 1 (TGF-β1) was increased in M2 macrophages (Fig. 2h). These data indicate that lncGBP9 and SOCS3 expression are upregulated in M1 macrophages and might be related to M1/2 polarization.

### Effects of lncGBP9 on macrophage polarization in vitro

**Fig. 3:**
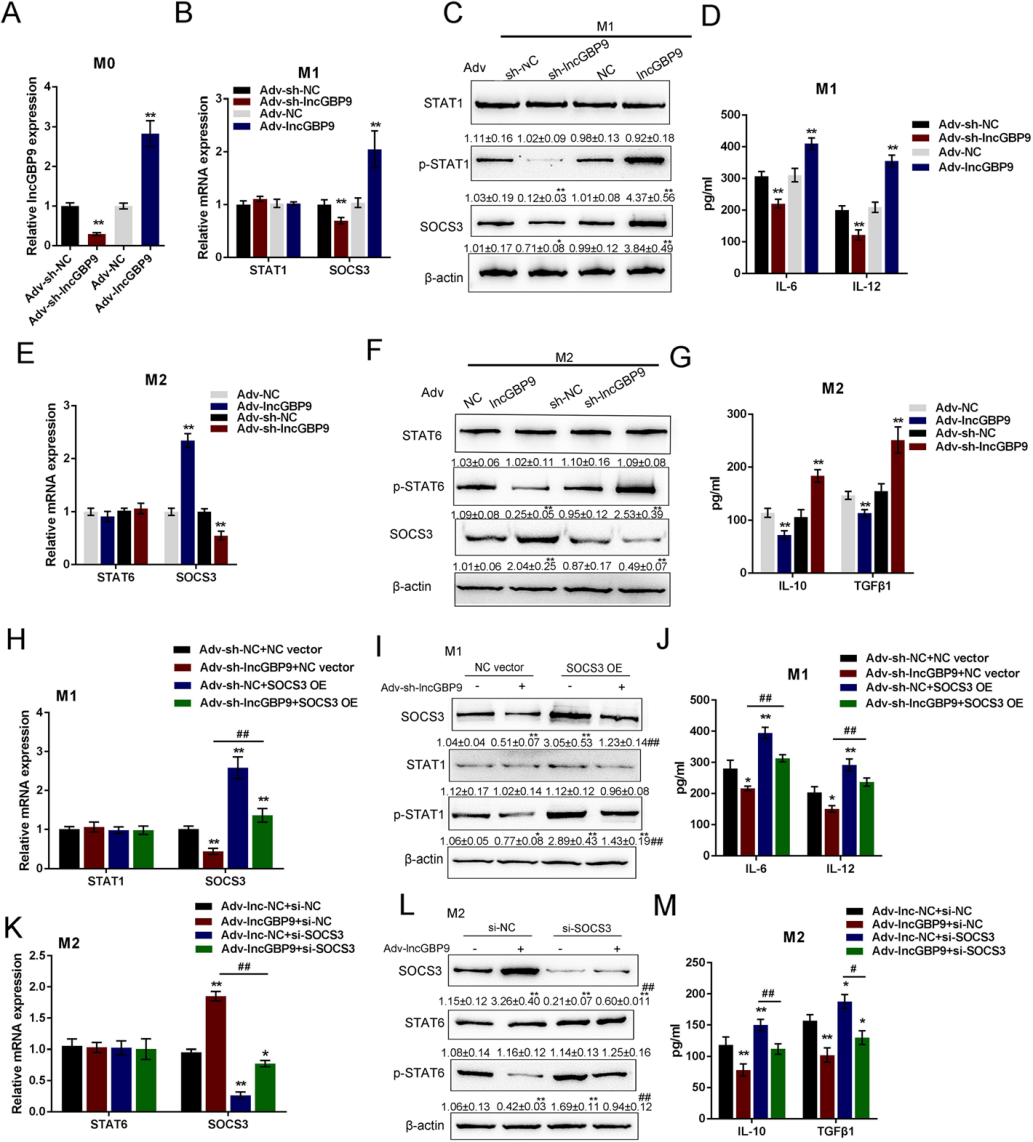
Effects of lncGBP9 on macrophage polarization in vitro. **a** BMDMs were induced toward M0 macrophages for 7 days as described in the M&M section. Then, the silencing and overexpression of lncGBP9 conducted in M0 macrophages for 48 h, as confirmed by real-time PCR. **b**, **c** Adv-sh-lncGBP9 or Adv-lncGBP9 transduced M0 macrophages were induced toward M1 polarization for 48 h and the mRNA expression and protein levels of STAT1, p-STAT1, and SOCS3 in response to lncGBP9 silencing or lncGBP9 overexpression were determined by real-time PCR and Immunoblotting in M1 macrophages. **d** The production of IL-6 and IL-12 in response to lncGBP9 silencing or lncGBP9 overexpression was determined by ELISA in M1 macrophages. **e, f** Adv-lncGBP9 or Adv-sh-lncGBP9 transduced M0 macrophages were induced toward M2 polarization for 48 h and the mRNA expression and protein levels of STAT6, p-STAT6, and SOCS3 in response to lncGBP9 overexpression or lncGBP9 silencing were determined by real-time PCR and Immunoblotting in M2 macrophages. **g** The production of IL-10 and TGF-β1 in response to lncGBP9 overexpression or lncGBP9 silencing was determined by ELISA in M2 macrophages. Next, M1 macrophages were co-transfected with Adv-lncGBP9 and SOCS3-overexpressing vector (SOCS3 OE) and examined for **h** the mRNA of STAT1 and SOCS3 by real-time PCR. **i** The protein levels of SOCS3, STAT1, and p-STAT1 by Immunoblotting. **j** The concentrations of IL-6 and IL-12 by ELISA. M2 macrophages were co-transfected with Adv-lncGBP9 and si-SOCS3 and examined for **k** the mRNA of STAT6 and SOCS3 by real-time PCR. **l** The protein levels of SOCS3, STAT6, and p-STAT6 by immunoblotting. **m** The concentrations of IL-10 and TGFβ1 by ELISA. *P < 0.05, **P < 0.01, compared to the control group; #*P* < 0.05, ##*P* < 0.01, compared to the Adv-lncGBP9 + NC (negative control) vector or Adv-lncGBP9 + si-NC (negative control) group. Values are mean ± S.D of *n* = 3 independent experiments

After selecting lncGBP9 for further experiments, we next evaluated its effects on macrophage polarization in vitro and in vivo. BMDMs were induced toward M0 macrophages for 7 days as described. Then, the silencing and overexpression of lncGBP9 were conducted in M0 macrophages for 48 h, and the transduction was effective and confirmed by real-time PCR (Fig. 3a). After transduction for 24 h, LncGBP9-silenced and lncGBP9-overexpressing M0 macrophages were stimulated by LPS + IFN-γ for M1 polarization. In M1 macrophages, lncGBP9 silencing or overexpression caused no significant changes in STAT1 mRNA expression; lncGBP9 silencing significantly downregulated, while lncGBP9 overexpression upregulated SOCS3 mRNA expression (Fig. 3b). In M1 macrophages, the protein levels of p-STAT1 and SOCS3 were reduced considerably by lncGBP9 silencing while increased by lncGBP9 overexpression (Fig. 3c); consistently, the production of IL-6 and IL-12 was also inhibited by lncGBP9 silencing while promoted by lncGBP9 overexpression in M1 macrophages (Fig. 3d). On the contrary, lncGBP9-overexpressing or lncGBP9-silenced macrophages were stimulated with IL-4 for M2 polarization. In M2 macrophages, lncGBP9 overexpression or silencing caused no significant changes in STAT6 mRNA expression; lncGBP9 overexpression significantly upregulated, while lncGBP9 silencing downregulated SOCS3 mRNA expression (Fig. 3e). In M2 macrophages, lncGBP9 overexpression decreased p-STAT6 and increased SOCS3 protein, while lncGBP9 silencing increased p-STAT6 and decreased SOCS3 (Fig. 3f); consistently, the production of IL-10 and TGF-β1 was suppressed by lncGBP9 overexpression while promoted by lncGBP9 silencing in M2 macrophages (Fig. 3g). These data indicate that lncGBP9 might modulate macrophage M1/2 polarization through affecting SOCS3 and the phosphorylation of STAT1/STAT6.

To further investigate the speculation, the study then co-transfected M0 macrophages with Adv-lncGBP9 and SOCS3-overexpressing vector (SOCS3 OE) or with Adv-lncGBP9 and si-SOCS3, induced transfected M0 macrophages toward M1 polarization, and examined for macrophage M1/2 polarization. In Adv-sh-lncGBP9 and SOCS3 OE co-transfected M1 macrophages, lncGBP9 silencing significantly inhibited SOCS3 mRNA expression (Fig. 3h), decreased SOCS3 protein level and STAT1 phosphorylation (Fig. 3i), and reduced the concentrations of IL-6 and IL-12 (Fig. 3j). SOCS3 overexpression in M1 macrophages exerted opposite effects. The effects of lncGBP9 silencing on M1 macrophages were significantly reversed by SOCS3 overexpression. Next, M0 macrophages were co-transfected with Adv-lncGBP9 and si-SOCS3, induced toward M2 polarization, and examined accordingly. In Adv-lncGBP9 and si-SOCS3 co-transfected M2 macrophages, lncGBP9 overexpression significantly upregulated SOCS3 mRNA expression (Fig. 3k), increased SOCS3 protein level, inhibited STAT6 phosphorylation (Fig. 3l), and reduced the concentrations of IL-10 and TGFβ (Fig. 3m). SOCS3 silencing in M2 macrophages exerted opposite effects. The effects of lncGBP9 silencing on M2 macrophages were significantly reversed by SOCS3 silencing.

### Effects of lncGBP9 on macrophage polarization in vivo

**Fig. 4:**
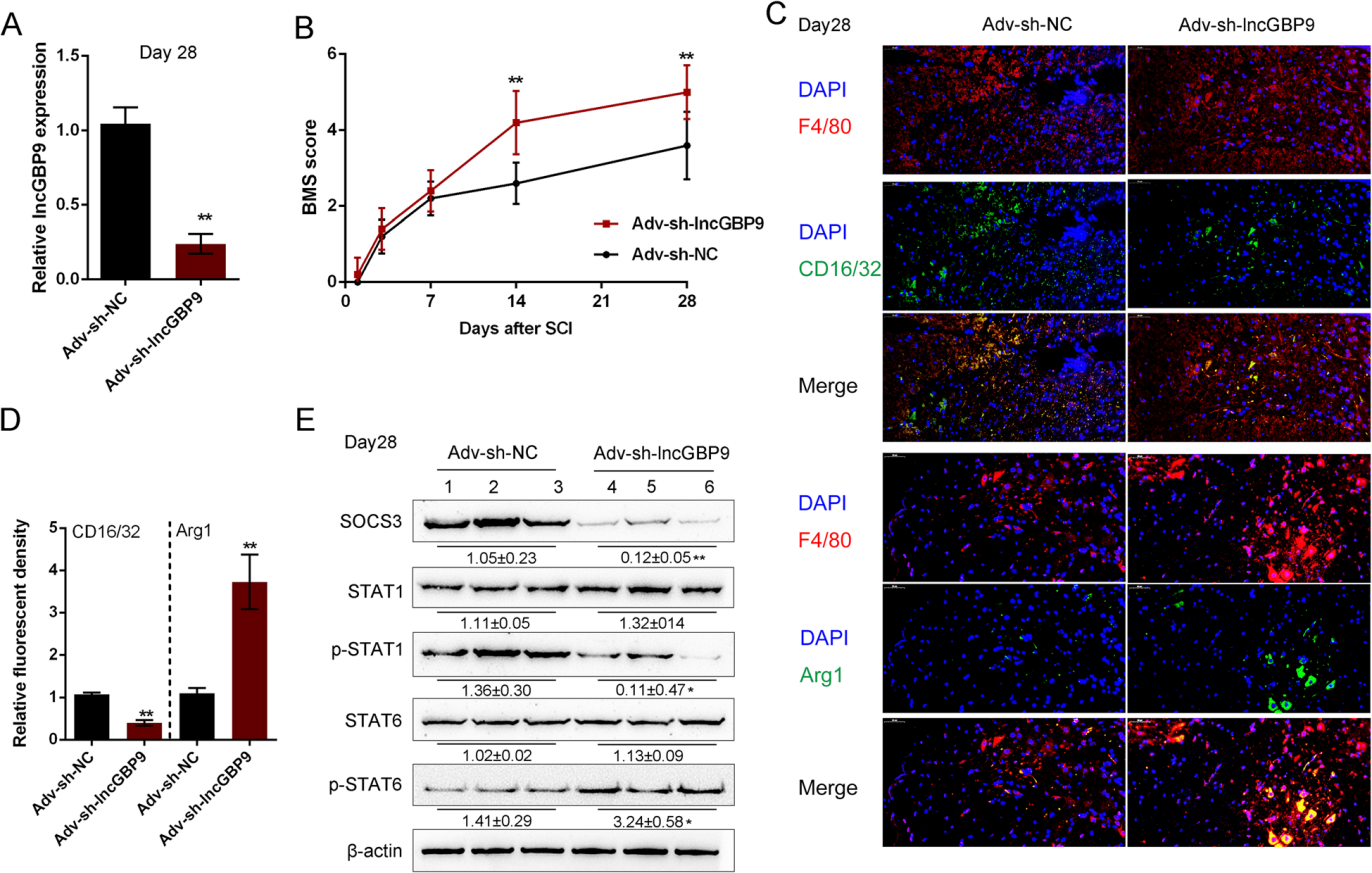
Effects of lncGBP9 on macrophage polarization in vivo. LncGBP9 silencing was conducted in the SCI mice model via tail vein injection Ad-sh-lncGBP9. *n* = 5 in each group. **a** The in vivo infection efficiency was verified by real-time PCR. **b** The BMS scores were evaluated on days 1, 3, 7, 14, and 28 after operation (*n* = 5). **c**, **d** The content and distribution of M1 macrophage marker CD16/32 and M2 macrophage marker Arg1 determined by IF staining on day 28 after operation (*n* = 5). **e** The protein levels of STAT1, p-STAT1, STAT6, p-STAT6, and SOCS3 determined by Immunoblotting on day 28 after operation (*n* = 3). **P < 0.01

Next, the effects of lncGBP9 on macrophage polarization were evaluated in vivo. LncGBP9 silencing was conducted in the SCI mice model via injecting to the epicenter of the injured spinal cord with Adv-sh-lncGBP9. On day 28 of SCI, Adv-sh-lncGBP9 effectively reduced the level of lncGBP9 in the injured spinal cords (Fig. 4a). Next, we evaluated the BMS scores on days 1, 3, 7, 14, and 28 after the operation to access the effects of lncGBP9 silencing on SCI severity. As shown in Fig. 4b, lncGBP9 silencing significantly increased the BMS scores in SCI mice on days 14 and 28 after the operation, indicating lncGBP9 silencing in SCI mice promoted the repair after SCI.

At the same time, the content and distribution of M1 macrophage marker CD16/32 and M2 macrophage marker Arg1 determined in lncGBP9-silenced SCI mice by IF staining and Immunoblotting on day 28 after the operation to investigate macrophage polarization. As shown in Fig. 4c, d, the fluorescence intensity representing M1 marker CD16/32 was significantly inhibited in Adv-sh-lncGBP9-infected mice on day 28, while M2 marker Arg1 was increased in Adv-sh-lncGBP9-infected mice on day 28, compared to those in Adv-sh-NC group. Consistently, Fig. 4e showed that the protein levels of p-STAT1 and SOCS3 were significantly decreased, while the protein levels of p-STAT6 were increased by lncGBP9 silencing in SCI mice on day 28 after the operation. These data indicate that lncGBP9 silencing might promote M2 and inhibit M1 polarization via STAT1/6 and SOCS3, therefore modulating the repair after SCI.

### LncGBP9 modulates SOCS3 through miR-34a in macrophages

LncRNAs could serve as ceRNAs for miRNAs to counteract miRNA-mediated suppression on miRNA downstream transcripts, therefore exerting their biological functions [33–35]. Essandoh reported a number of miRNAs that might promote M2 polarization [56]; among them, miR-124 and miR-34a were predicted to target SOCS3 and only miR-34a was predicted to target lncGBP9. More importantly, miR-34a could promote M2 macrophage polarization [40]. Thus, we hypothesize that miR-34a might participate in lncGBP9 function on macrophage polarization.

**Fig. 5:**
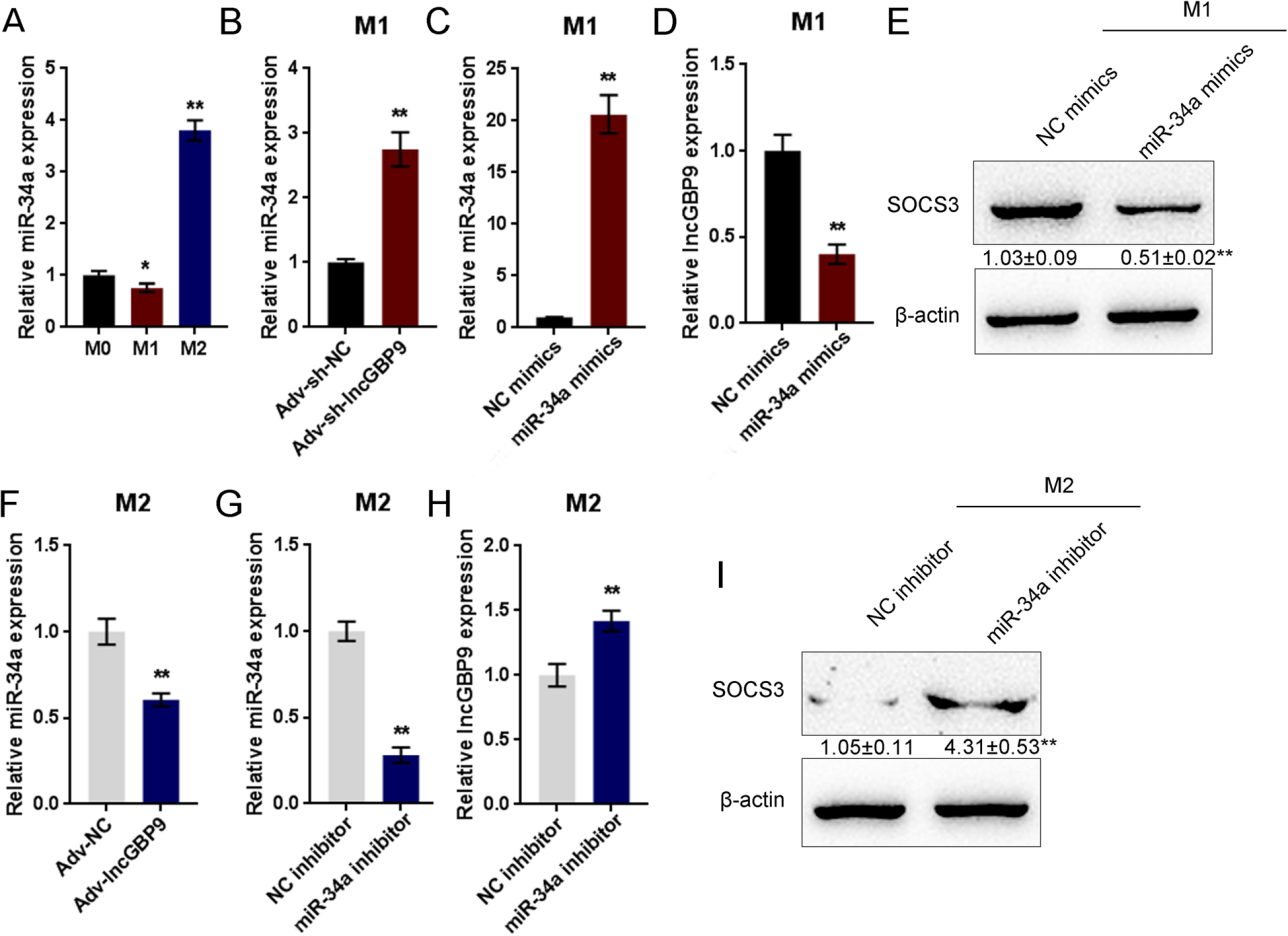
LncGBP9 modulates SOCS3 through miR-34a in macrophages. **a** The expression of miR-34a determined in M0, M1, and M2 macrophages by real-time PCR. **b** The expression of miR-34a in response to lncGBP9 silencing determined in M1 macrophages by real-time PCR. **c** miR-34a overexpression conducted in M1 macrophages by transfection of miR-34a mimics, as confirmed by real-time PCR. **d** LncGBP9 expression in response to miR-34a overexpression determined in M1 macrophages by real-time PCR. **e** SOCS3 protein level in response to miR-34a overexpression determined in M1 macrophages by immunoblotting. **f** miR-34a expression in response to lncGBP9 overexpression determined in M2 macrophages by real-time PCR. **g** miR-34a inhibition conducted in M2 macrophages by transfection of miR-34a inhibitor, as confirmed by real-time PCR. **h** LncGBP9 expression in response to miR-34a inhibition determined in M2 macrophages by real-time PCR. **i** SOCS3 protein level in response to miR-34a inhibition determined in M2 macrophages by Immunoblotting. *P < 0.05, **P < 0.01. Values are mean ± S.D of *n* = 4 independent experiments

To validate the hypothesis, we examined the expression of miR-34a in vivo and in vitro, including in M0, M1, and M2 macrophages. In SCI mice, miR-34a expression was significantly downregulated on day 28 after the operation (Fig. S2A); in SCI mice infected with Adv-sh-lncGBP9, miR-34a expression was significantly upregulated on day 28 after the operation, compared to Adv-sh-NC group (Fig. S2B). As shown in Fig. 5a, miR-34a expression was dramatically upregulated in M2 macrophages. In M1 macrophages, miR-34a expression was significantly increased by lncGBP9 silencing (Fig. 5b). To investigate the cellular effects of miR-34a, we conducted miR-34a overexpression in M1 macrophages by transfection of miR-34a mimics into M0 macrophages before polarization, as confirmed by real-time PCR (Fig. 5c). LncGBP9 expression was significantly downregulated by miR-34a overexpression in M1 macrophages (Fig. 5d). Consistently, the SOCS3 protein level was also decreased by miR-34a overexpression in M1 macrophages (Fig. 5e).

In M2 macrophages, miR-34a expression was significantly downregulated by lncGBP9 overexpression (Fig. 5f). Here, we conducted miR-34a inhibition M2 macrophages by transfection of miR-34a inhibitor, as confirmed by real-time PCR (Fig. 5g). In M2 macrophages, lncGBP9 expression was significantly upregulated by miR-34a inhibition (Fig. 5h). Consistently, the SOCS3 protein level was increased by miR-34a inhibition in M2 macrophages (Fig. 5i). These data indicate that lncGBP9 might regulate SOCS3 through miR-34a to participate in M1/2 macrophage polarization.

### LncGBP9 serves as a ceRNA for miR-34a to counteract miR-34a-mediated SOCS3 suppression

**Fig. 6:**
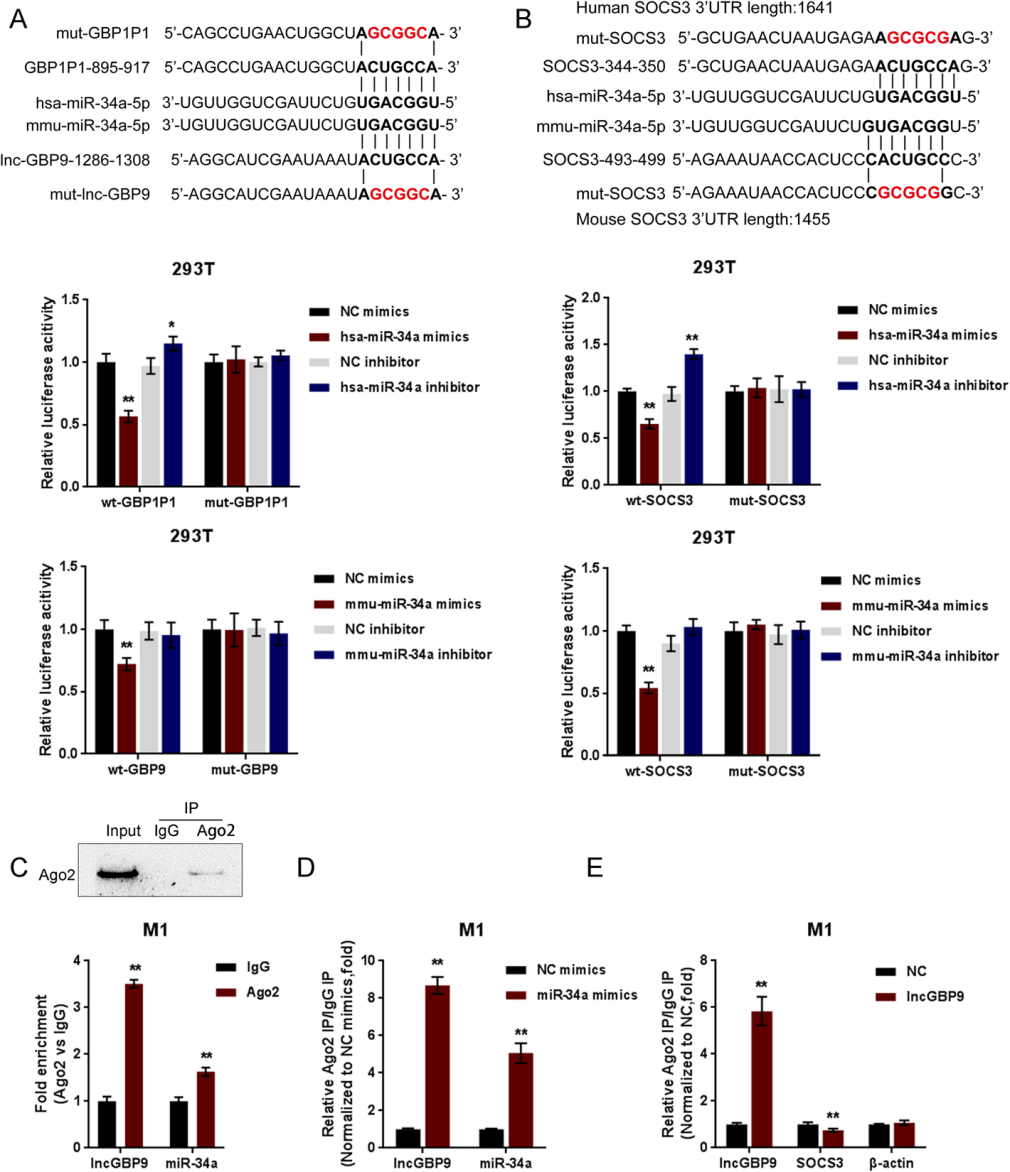
LncGBP9 serves as a ceRNA for miR-34a to counteract miR-34a-mediated SOCS3 suppression. **a**, **b** Schematic diagrams showing the predicted binding site between miR-34a and lncGBP9 and between miR-34a and SOCS3. Wild- and mutant-type GBP9 and SOCS3 3′-UTR luciferase reporter vectors (wt-GBP9/SOCS3 3′-UTR or mut-GBP9/SOCS3 3′-UTR) were constructed and co-transfected in 293T cells with miR-34a mimics/inhibitor; the luciferase activity was determined. **c** Association of miR-34a and lncGBP9 with AGO2 in M1 macrophages. Detection of AGO2 and IgG using Immunoblotting assays. **d** RIP assay in M1 macrophages transfected with control miRNA (NC mimics) or miR-34a mimics followed by real-time PCR to detect GBP9 and miR-34a associated with AGO2. IgG was used as negative control. **e** RIP assay in M1 macrophages transfected with control vector (NC) or lncGBP9-overexpressing vector followed by real-time PCR to detect GBP9, SOCS3, and β-actin associated with AGO2. β-actin was used as negative control. *P < 0.05, **P < 0.01. Values are mean ± S.D of *n* = 3 independent experiments

To validate the predicted targeting of miR-34a to lncGBP9 and SOCS3, we performed luciferase reporter assays by constructing wild- and mutant-type GBP9 and SOCS3 3′-UTR luciferase reporter vectors (wt-GBP9/SOCS3 3′-UTR or mut-GBP9/SOCS3 3′-UTR) as described in M&M section (Fig. 6a, b). Next, 293T cells were co-transfected with the above-described vectors and miR-34a mimics/inhibitor and examined for the luciferase activity. As shown in Fig. 6a, b, the luciferase activity of wt-GBP9 and wt-SOCS3 3′-UTR vectors could be significantly inhibited by miR-34a overexpression and enhanced by miR-34a inhibition; in responding to the mutation at the putative miR-34a binding sites, the changes in the luciferase activity were abolished. Moreover, in the RNA derived from precipitated AGO2 protein, lncGBP9 and miR-34a levels were significantly higher than those in IgG in M1 macrophages (Fig. 6c). We also performed RIP assay in M1 macrophages transfected with NC mimics or miR-34a mimics and then detected lncGBP9 and miR-34a levels associated with AGO2; the results shown in Fig. 6d confirmed the interaction between lncGBP9 and miR-34a. Furthermore, in lncGBP9-overexpressing M1 macrophages, the level of lncGBP9 detected was dramatically higher than that of NC group. While the levels of SOCS3 was lower than that of NC group (Fig. 6e), indicating that lncGBP9 and SOCS3 could bind miR-34a, respectively; lncGBP9 competes with SOCS3 for miR-34a binding.

### LncGBP9/miR-34a axis modulates macrophage polarization via affecting the balance of STAT1/STAT6

**Fig. 7:**
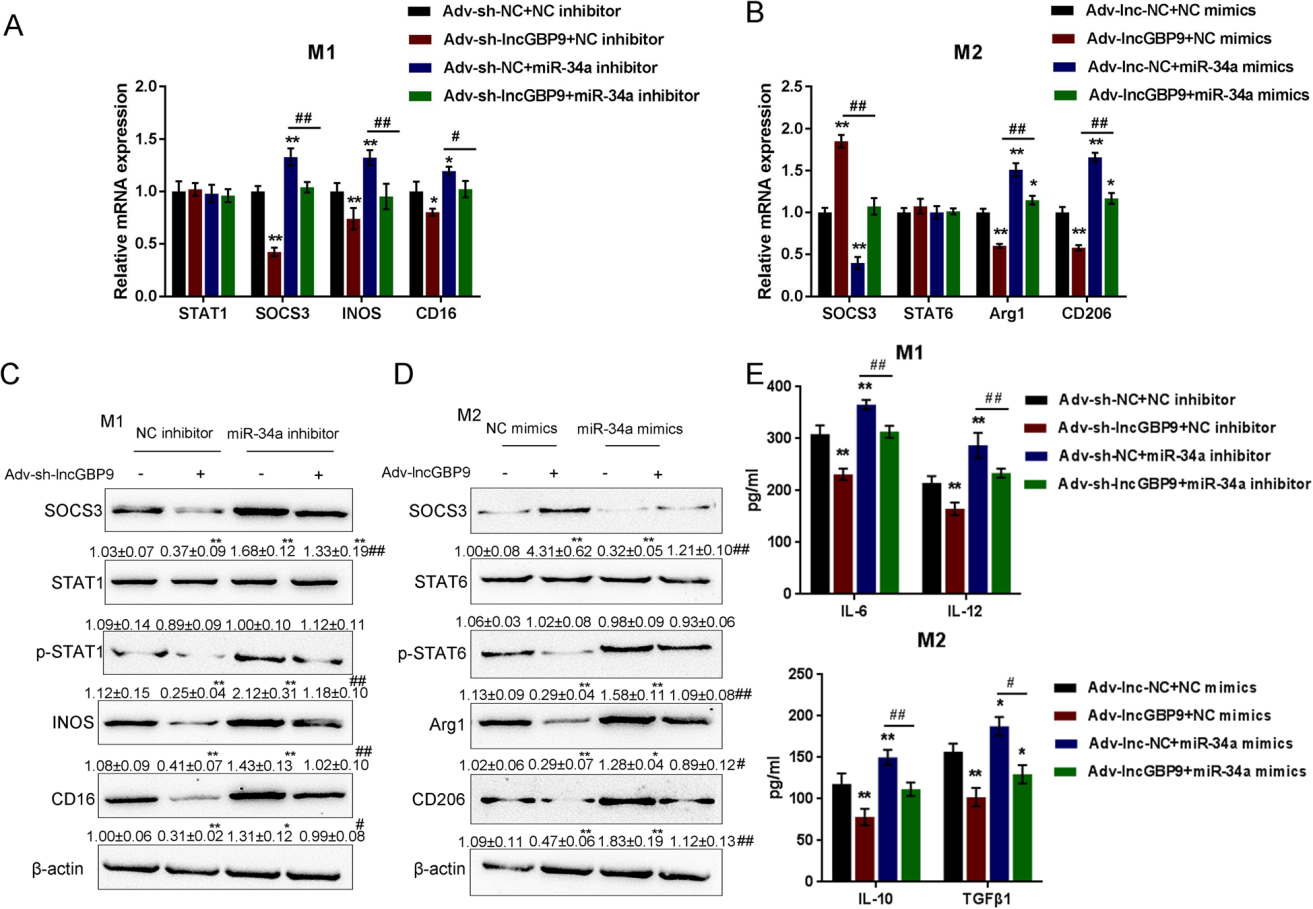
LncGBP9/miR-34a axis modulates macrophage polarization via affecting the balance of STAT1/STAT6. **a**, **c**, **e** M1 macrophages were co-transfected with Ad-sh-lncGBP9 and miR-34a inhibitor and examined for the mRNA expression and protein levels of STAT1, p-STAT1, SOCS3, iNOS, and CD16/32, and the production of IL-6 and IL-12. **b**, **d**, **e** M2 macrophages were co-transfected with Ad-lncGBP9 and miR-34a mimics and examined for the mRNA expression and protein levels of STAT6, p-STAT6, SOCS3, Arg1, and CD206, and the production of IL-10 and TGF-β1. *P < 0.05, **P < 0.01, compared to control group; #*P* < 0.05, ##*P* < 0.01, compared to Ad-sh-NC + miR-34a inhibitor or Ad-lnc-NC + miR-34a mimics group. Values are mean ± S.D of *n* = 3 independent experiments

After confirming the binding of miR-34a to lncGBP9 and SOCS3, next, we evaluated the dynamic effects of lncGBP9 and miR-34a on STAT1/STAT6 and macrophage polarization. M0 macrophages were co-transfected with Ad-sh-lncGBP9 and miR-34a inhibitor and then polarized to M1 macrophages; the mRNA expression and protein levels of STAT1, p-STAT1, SOCS3, iNOS, and CD16, and the production of IL-6 and IL-12 were examined. As shown in Fig. 7a, c, e, lncGBP9 silencing significantly reduced, while miR-34a inhibition significantly increased the mRNA expression and protein levels of p-STAT1, SOCS3, iNOS, and CD16/32, as well as the production of IL-6 and IL-12 in M1 macrophages; the effects of lncGBP9 silencing could be significantly reversed by miR-34a inhibition.

M0 macrophages were co-transfected with Ad-lncGBP9 and miR-34a mimics and then polarized to M2 macrophages, the mRNA expression and protein levels of STAT6, p-STAT6, SOCS3, Arg1, and CD206, and the production of IL-10 and TGF-β1 were examined. As shown in Fig. 7b, d, e, lncGBP9 overexpression significantly increased SOCS3 mRNA expression and protein level; decreased p-STAT6, Arg1, and CD206 mRNA and protein levels; and suppressed the production of IL-10 and TGF-β1. miR-34a overexpression exerted opposing effects on these indicators; the effects of lncGBP9 overexpression could be significantly reversed by miR-34a overexpression. These data indicate that the lncGBP9/miR-34a axis modulates M1/2 macrophage polarization through SOCS3 and STAT1/STAT6.

### STAT6 binds miR-34a promoter to activate its transcription

**Fig. 8:**
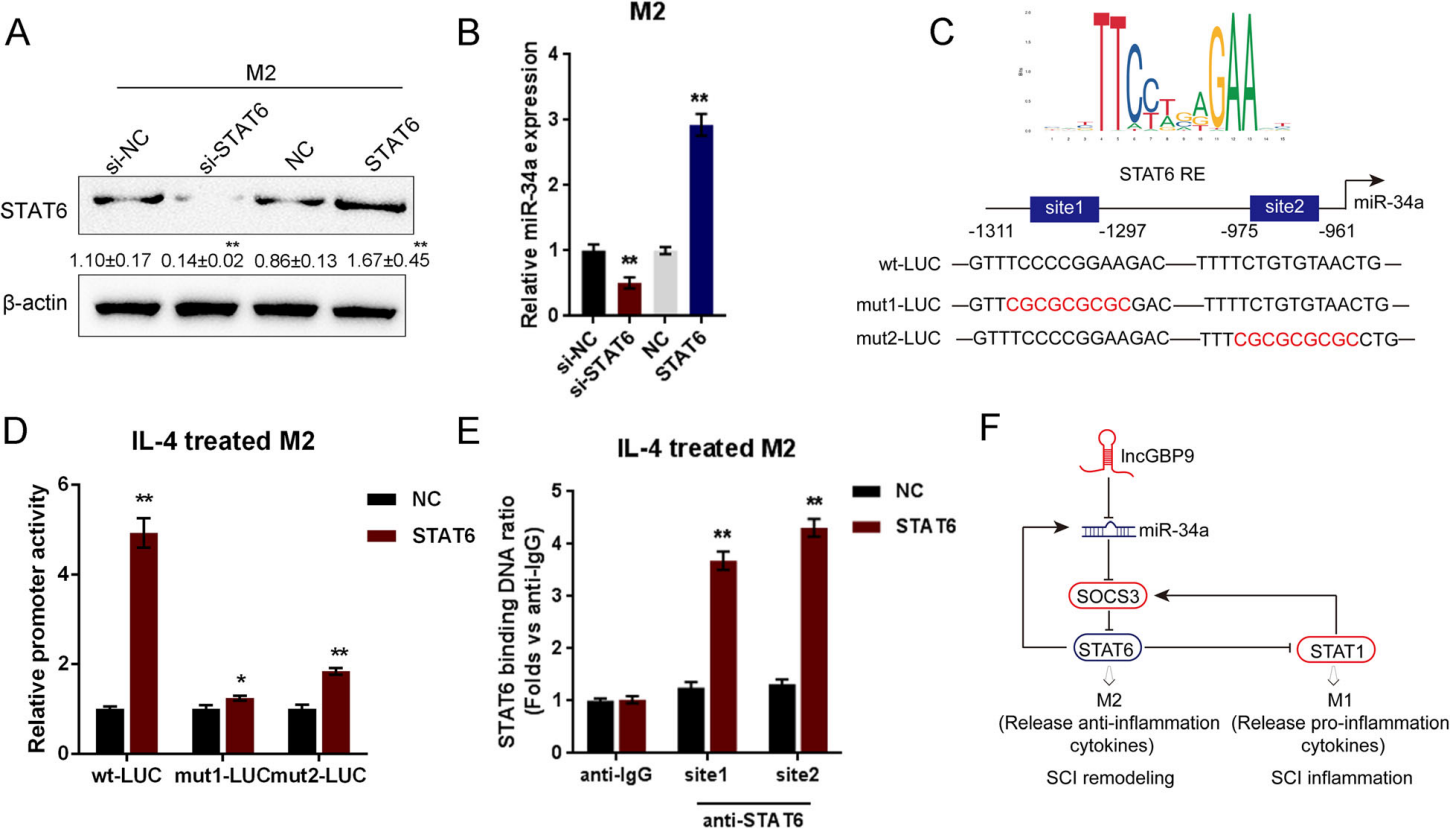
STAT6 binds miR-34a promoter to activate its transcription. **a** STAT6 overexpression or silencing conducted in M2 macrophages by transfection of STAT6-overexpressing or si-STAT6 vector into M0 and then polarizing to M2, as confirmed by Immunoblotting. **b** The expression of miR-34a in STAT6-overexpressing or STAT6-silenced M2 macrophages determined by real-time PCR. **c** A schematic diagram showing the predicted binding sites between STAT6 and miR-34a promoter. Wild- and mutant-type miR-34a luciferase reporter vectors are constructed. **d** STAT6 and wt- or mut-miR-34a were co-transfected in M0 macrophages followed M2 polarization; the luciferase activity was determined. **e** The real-time ChIP assay showed that the level of STAT6 antibody binding to miR-34a promoter was much greater than that of IgG. **f** In macrophages, lncGBP9 competed with SOCS3 for miR-34a binding to counteract miR-34a-mediated suppression on SOCS3, therefore modulating STAT1/STAT6 signaling and the polarization of macrophages. STAT6 bound the promoter of miR-34a to activate its transcription, therefore forming two different regulatory loops to modulate the polarization of macrophages after SCI. Values are mean ± S.D of *n* = 3 independent experiments

As predicted by the online tool, STAT6 might bind the promoter region of miR-34a to activate its transcription. STAT6 overexpression or silencing was conducted in M0 macrophages by transfection of STAT6-overexpressing or si-STAT6 vector following M2 polarization, as confirmed by immunoblotting (Fig. 8a). In M2 macrophages, the expression of miR-34a was significantly upregulated by STAT6 overexpression while downregulated by STAT6 silencing (Fig. 8b). Next, wild- and mutant-type miR-34a luciferase reporter vectors are constructed; the mut-miR-34a vector contained a 9-bp mutation in any of the predicted STAT6 binding sites (Fig. 8c). STAT6 and wt- or mut-miR-34a promoter were then co-transfected in M0 macrophages followed by M2 polarization; the luciferase activity was determined. As shown in Fig. 8d, the promoter activity of wt-miR-34a was dramatically increased by STAT6 overexpression; however, after mutating any of the predicted binding sites, STAT6 overexpression-induced increase in promoter activity was abolished (Fig. 8d). Moreover, the ChIP assay showed that the level of STAT6 antibody binding to miR-34a binding element in the miR-34a promoter was much greater than that of IgG in M2 macrophages (Fig. 8e), suggesting that STAT6 might bind to the promoter of miR-34a to activate its expression in M2 macrophages.

## Discussion

In the present study, we constructed the SCI model in Balb/c mice and observed that the macrophages in the spinal cord were skewed toward M1 phenotype after SCI. BMDMs were isolated and identified. LncRNA GBP1P1 (lncGBP9 in mice) has been previously reported to be upregulated and observed overexpressed in M1 macrophages in the present study. SOCS3 and p-STAT1, key factors in macrophage polarization, were also overexpressed in M1 and underexpressed in M2 macrophages while p-STAT6 was underexpressed in M1 and overexpressed in M2 macrophages. Consistently, IL-6 and IL-12 were increased in M1 while IL-10 and TGF-β1 were increased in M2 macrophages. In M1 macrophages, lncGBP9 silencing significantly decreased p-STAT1 and SOCS3 expression and protein levels, as well as the production of IL-6 and IL-12; in M2 macrophages, lncGBP9 overexpression increased SOCS3 expression and protein levels while suppressed p-STAT6 levels and the production of IL-10 and TGF-β1, indicating that lncGBP9 overexpression promotes the M1 polarization of macrophages. In lncGBP9-silenced SCI mice, the BMS scores were significantly higher from day 14 after the operation, and the M2 polarization was promoted on day 28 after the operation, further indicating that lncGBP9 silencing revised the predominance of M1 phenotype at the late stage of secondary injury after SCI, therefore improving the repair after SCI. In macrophages, lncGBP9 competed with SOCS3 for miR-34a binding to counteract miR-34a-mediated suppression on SOCS3, therefore modulating STAT1/STAT6 signaling and the polarization of macrophages. Finally, STAT6 bound the promoter of miR-34a to activate its transcription, therefore forming two different regulatory loops to modulate the polarization of macrophages after SCI (Fig. 8f).

As mentioned earlier, during the occurrence and development of SCI, via enhanced phagocytosis and increased production and release of pro-inflammatory cytokines, M1 macrophages promote innate immunity to remove foreign microorganisms and wound fragments from damaged sites. Differently, M2 macrophages have tissue repair properties, showing a decrease in inflammatory cytokines and in the production of ROS [57, 58]. These stimuli induce M2 macrophages to regulate inflammatory reactions, remove debris, and facilitate tissue remodeling and repair. This sequential M1-M2 macrophage response will lead to successful SCI injury repair [59]. That is, the skewing macrophage toward the M1 phenotype may cause SCI repair to fail. At the early phase after the injury, the production and release of certain inflammatory cytokines would be induced by macrophages [44, 60]. During the early stage of SCI in the SCI mice model, Kigerl et al. [45] reported that M1 macrophages accounted for the majority. After SCI, M1 and M2 biomarkers all increased rapidly; however, Arg1, one of the M2 biomarkers, was only transient and reverted to the baseline 7 days later post-SCI [45]. On the 14th day after SCI, CD206, another M2 biomarker, was significantly increased in comparison to the normal spinal cord tissue. Differently, M1 marker iNOS transient increased until day 3 after the injury; CD32, another M1 marker, significantly decreased on day 28 post-SCI. It appears that the expression of iNOS and Arg1 is regulated by each other, suggesting that not all M1 and M2 biomarkers change their expression in a coordinated manner after SCI, possibly because the regulation of macrophage polarization begins at a different time and different phases post-SCI, or the inflammatory microenvironment affects these factors in different manners. In the present study, we observed the levels of M1 markers, including iNOS, CD16/32, and IFN-γ, increased after SCI from day 3 to day 28 in a time-dependent manner. On the contrary, the expression of M2 markers, Arg1, CD206, and IL-4, reached peak values on day 7 or 14 while decreased on day 28 after SCI. These findings indicate that the predominance of M1 macrophages continues to the late phase after SCI. The skewing macrophage toward the M1 might contribute to the failure of SCI repair.

As we have mentioned, the polarization of macrophages could be regulated by different stimuli and factors, including protein-coding and non-coding RNAs. The genes related to the macrophage polarization might, in turn, contribute to the dysfunction of macrophages. Huang et al. analyzed the differentially expressed genes in M0, M1, and M2 macrophages and demonstrated that 2528 mRNAs were overexpressed and 4534 mRNAs were underexpressed in the M2 group compared with the M1 group. More importantly, there was a significant steady-state in the expression levels of 275 mRNAs between three groups [38]. Another group identified a total of 1253 differentially expressed genes between M1 and M2 macrophages, of which 696 were upregulated and 557 downregulated in M1 macrophages compared with M2 macrophages [55]. Based on these previous findings, we performed protein-protein interaction analysis and revealed that TNF, SOCS3, and STAT1 were at the core location of macrophage polarization. SOCS proteins are a family of eight intracellular cytokine-inducible proteins [61, 62] obtaining a basal expression in cells. SOCSs could be sharply induced by many stimuli, including cytokines, TLR ligands, immune complexes, and hormones [63]. Although SOCSs are expressed at a very low level in macrophages, they could also be rapidly induced upon activation. SOCS1 and SOCS3 can regulate the polarization of macrophages to M1 and/or M2 subtypes [64, 65]. More importantly, IFN-γ/STAT1, IL-4/STAT6, and IL-12/STAT4 signaling pathways in differentiating Th cells may be under feedback regulation by SOCS [66–68]. Yu et al. [69] revealed that in STAT1^−/−^ Th2 cells, SOCS1 and SOCS3 protein levels are remarkably reduced; besides, they also demonstrated that SOCS1 and SOCS3 could lead to the suppression of STAT6 signaling. In the present study, we observed that the phosphorylation of STAT1 dramatically increased in M1 macrophages while STAT6 phosphorylation increased in M2 macrophages. Consistently, SOCS3 expression was upregulated in both M1 and M2 macrophages compared to M0 type but was significantly higher in M1 compared to M2 macrophages. These findings suggest that STAT1/STAT6 signaling and SOCS3 might participate in the progress of macrophage polarization. Regarding differentially expressed lncRNAs, lncRNA GBP1P1 (lncGBP9 in mice) was significantly upregulated in M1 macrophages according to previous studies (GSE117040 and GSE5099) and our observations, suggesting that lncGBP9 may play a role in M1/M2 macrophage polarization.

As we have mentioned, GBP1P1 is a pseudogene of the guanylate-binding protein of GBP. GBPs account for over 20% of the proteins induced after IFN-γ treatment [70, 71]. GBP family is also involved in macrophage functions, such as IFN-γ-mediated macrophage activation and immune defense [54]. More importantly, lncRNA GBP1P1 expression was significantly upregulated in M1 macrophages according to several online data (Fig. S1). In the present study, by conducting lncGBP9 silencing in M1 macrophages, we observed a decreased expression of SOCS3 and suppressed phosphorylation of STAT1, as well as reduced production of IL-6 and IL-12. On the contrary, lncGBP9 overexpression in M2 macrophages significantly induced the upregulation of SOCS3 while suppressed the phosphorylation of STAT6 and the production of IL-10 and TGF-β1. Notably, the effects of lncGBP9 silencing on M1 macrophages were significantly reversed by SOCS3 overexpression while the effects of lncGBP9 overexpression on M2 macrophages were significantly reversed by SOCS3 silencing. Consistently, in SCI mice model, lncGBP9 silencing significantly suppressed STAT1 phosphorylation and SOCS3 expression while promoted STAT6 phosphorylation on day 28 after SCI; in the meantime, lncGBP9 silencing caused a significant decrease in the BMS scores, indicating that lncGBP9 silencing inhibits SOCS3 while promotes STAT6 activation at the late phase of SCI, therefore improving the SCI repair.

It has recently been discovered that lncRNAs act as miRNA “sponges” by sharing common miRNAs responses elements (MRE) and inhibiting the targeting activity of miRNAs on downstream target mRNAs, therefore forming posttranscriptional ceRNA networks to regulate the expression of downstream target mRNAs and participating in biological processes [34]. We have revealed that lncGBP9 plays an essential role in M1/M2 macrophage polarization via SOCS3 and STAT1/STAT6. Here, we hypothesize that miRNAs might mediate the function of lncGBP9 in macrophage polarization. miR-34a, previously known for its potent tumor-suppressive role, has been regarded as an inflammation regulator. Jiang et al. [40] reported that the expression of miR-34a was downregulated in macrophages after LPS stimulation. MiR-34a overexpression decreased the expression of inflammatory cytokines TNF-α and IL-6 in LPS-treated RAW264.7 cells. Furthermore, LPS-induced NF-κB activation was also significantly suppressed by miR-34a. In the present study, online tools predicted that miR-34a might target both lncGBP9 and SOCS3. Consistent with the previous studies, miR-34a expression was significantly downregulated in M1 macrophages after LPS + IFN-γ stimulation while upregulated in M2 macrophages. miR-34a overexpression in M1 macrophages significantly inhibited lncGBP9 expression and reduced SOCS3 protein levels; on the contrary, miR-34a inhibition in M2 macrophages promoted the expression of lncGBP9 and the protein levels of SOCS3. Regarding the molecular mechanism, miR-34a directly targets lncGBP9 and SOCS3 3′-UTR. LncGBP9 competed with SOCS3 for miR-34a binding, therefore abolishing miR-34a-mediated SOCS3 suppression. LncGBP9 silencing significantly decreased the levels of SOCS3 and M1 macrophage markers, while lncGBP9 overexpression increased SOCS3 while reduced the levels of M2 macrophage markers. In both macrophage types, the effects of miR-34a were opposite to those of lncGBP9. More importantly, the effects of lncGBP9 could be significantly reversed by miR-34a, indicating that lncGBP9 exerts its functions in macrophage polarization via serving as a ceRNA for miR-34a to counteracting miR-34a-mediated SOCS3 suppression.

Interestingly, the phosphorylation of STAT6 and miR-34a expression is significantly upregulated in M2 macrophages. Previously, STAT6 has been regarded as the dominant mediator in IL-4-induced transcriptional alterations in macrophages [72]; herein, we speculated that STAT6 might also take responsibility for the IL-4-induced miR-34a upregulation in macrophages. As predicted by online tools and later confirmed by luciferase reporter and ChIP assays, STAT6 binds the promoter region of miR-34a to activate its transcription.

## Conclusions

In summary, in macrophages, lncGBP9 competed with SOCS3 for miR-34a binding to counteract miR-34a-mediated suppression on SOCS3, therefore modulating STAT1/STAT6 signaling and the polarization of macrophages. STAT6 bound the promoter of miR-34a to activate its transcription, therefore forming two different regulatory loops to modulate the polarization of macrophages after SCI (Fig. 8f). We provide a novel strategy for improving the failure in SCI repair.

## Supplementary information

**Additional file 1: Figure S1**. RNA-seq analyses GSE5099, GSE117040, E-GEOD-57494, E-MTAB-2399 and GSE40885 (A) A schematic diagram showing the process of selecting lncRNAs upregulated in M1 macrophages based on GSE117040 and GSE5099. LncRNA GBP1P1 (GBP9 in mice) and LINC00869 (Fam91a1 in mice) were selected after cross-check and literature review. (B) GSE5099 reported differentially-expressed genes at different time point during inducing the monocytes into macrophages. LncRNA GBP1P1 is significantly up-regulated in M1 macrophages compared to that in monocytes and M2 macrophages; (C) GSE117040 performed RNA-Seq analysis on RNA expression in M1 and M2 polarized human macrophages (4 replicate samples) and showed that lncRNA GBP1P1 expression was significantly up-regulated in M1 macrophages; (D) E-GEOD-57494 performed RNA-Seq analysis on RNA expression in human monocytes (cd14+/cd16+) treated with LPS + IFN-γ, compared to DMSO treatment group. LncRNA GBP1P1 was significantly upregulated in LPS + IFN-γ group 6 hours or 24 hours after the LPS + IFN-γ treatment; (E) E-MTAB-2399 performed RNA-Seq analysis on RNA expression in human monocytes subjected to 10 ng/ml LPS treatment. LncRNA GBP1P1 was rapidly upregulated by LPS treatment; (F) GSE40885 performed RNA-Seq analysis on RNA expression in human alveolar macrophages induced by LPS. LncRNA GBP1P1 was rapidly upregulated by LPS treatment.

**Additional file 2: Figure S2**. In vivo expression level of miR-34a in SCI mice (A) or Adv-sh-lncGBP9 infected SCI mice at day 28 of SCI treatment (B). Values are mean ± S.D of n = 5 independent experiments.

**Additional file 3: Table S1**. The primer sequence

## Data Availability

Please contact the authors for data requests.

## Abbreviations

BMDMs: Bone marrow-derived macrophages
ChIP: Chromatin immunoprecipitation
CNS: Central nervous system
lncRNAs: Long non-coding RNAs
miRNAs: MicroRNAs
PGE2: Prostaglandin
RIP: RNA immunoprecipitation
RNS: Reactive nitrogen
ROS: Reactive oxygen species
SCI: Spinal cord injury
SOCS3: Suppressor of cytokine signaling 3
STAT1: Signal transducer and activator of transcription 1
STAT6: Signal transducer and activator of transcription 6
Arg-1: Arginase
MRE: MiRNAs responses elements
STRING: Search tool for the retrieval of interacting genes/protein
GBP: Guanylate-binding protein
IL: Interleukin
TGF-β1: Transforming growth factor-beta 1
TH1: Prototypical T-helper 1 cytokine
IFN-γ: Interferon-γ
CCL5: C-C motif chemokine ligand 5
TLRs: Toll-like receptors
MyD88: Myeloid differentiation factor 88
NF-κB: Nuclear factor kappa B
JAK: Janus kinase
JNK: c-Jun N-terminal kinases
MAPK: Mitogen-activated protein kinase
PI3K: Phosphoinositide 3-kinases
Akt: Protein kinase B
PAK1: P21 (RAC1) activated kinase 1
