# Supplementary figures and images for: LncGBP9/miR-34a axis drives macrophages toward a phenotype conducive for spinal cord injury repair via STAT1/STAT6 and SOCS3
Source: J Neuroinflammation. 2020 Apr 28;17:134. doi: 10.1186/s12974-020-01805-5 (PMC7187522; doi:10.1186/s12974-020-01805-5)

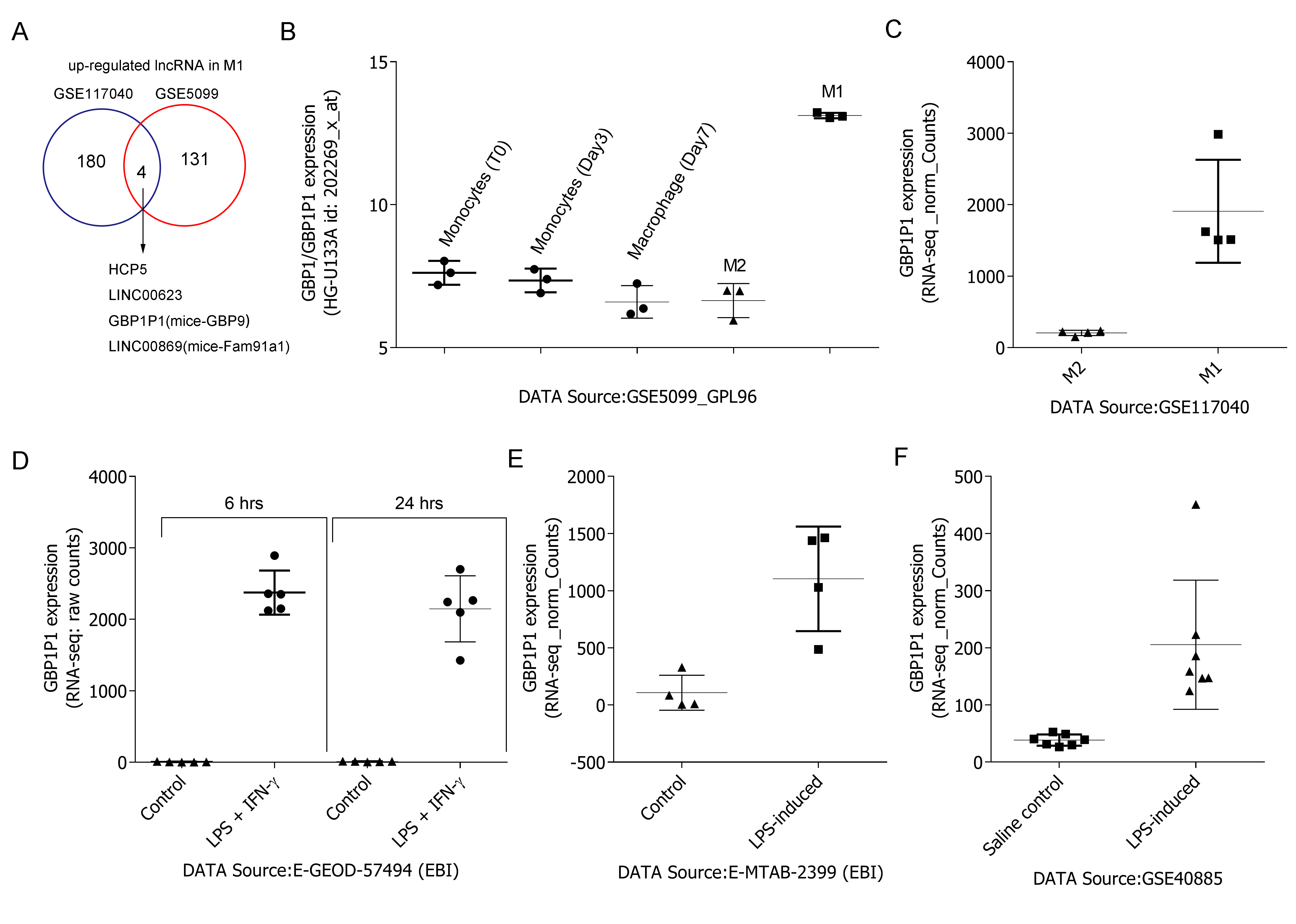

Supplement: Supplementary file 1 — Additional file 1: Figure S1. RNA-seq analyses GSE5099, GSE117040, E-GEOD-57494, E-MTAB-2399 and GSE40885 (A) A schematic diagram showing the process of selecting lncRNAs upregulated in M1 macrophages based on GSE117040 and GSE5099. LncRNA GBP1P1 (GBP9 in mice) and LINC00869 (Fam91a1 in mice) were selected after cross-check and literature review. (B) GSE5099 reported differentially-expressed genes at different time point during inducing the monocytes into macrophages. LncRNA GBP1P1 is significantly up-regulated in M1 macrophages compared to that in monocytes and M2 macrophages; (C) GSE117040 performed RNA-Seq analysis on RNA expression in M1 and M2 polarized human macrophages (4 replicate samples) and showed that lncRNA GBP1P1 expression was significantly up-regulated in M1 macrophages; (D) E-GEOD-57494 performed RNA-Seq analysis on RNA expression in human monocytes (cd14+/cd16+) treated with LPS + IFN-γ, compared to DMSO treatment group. LncRNA GBP1P1 was significantly upregulated in LPS + IFN-γ group 6 hours or 24 hours after the LPS + IFN-γ treatment; (E) E-MTAB-2399 performed RNA-Seq analysis on RNA expression in human monocytes subjected to 10 ng/ml LPS treatment. LncRNA GBP1P1 was rapidly upregulated by LPS treatment; (F) GSE40885 performed RNA-Seq analysis on RNA expression in human alveolar macrophages induced by LPS. LncRNA GBP1P1 was rapidly upregulated by LPS treatment. [file 12974_2020_1805_MOESM1_ESM.tif]

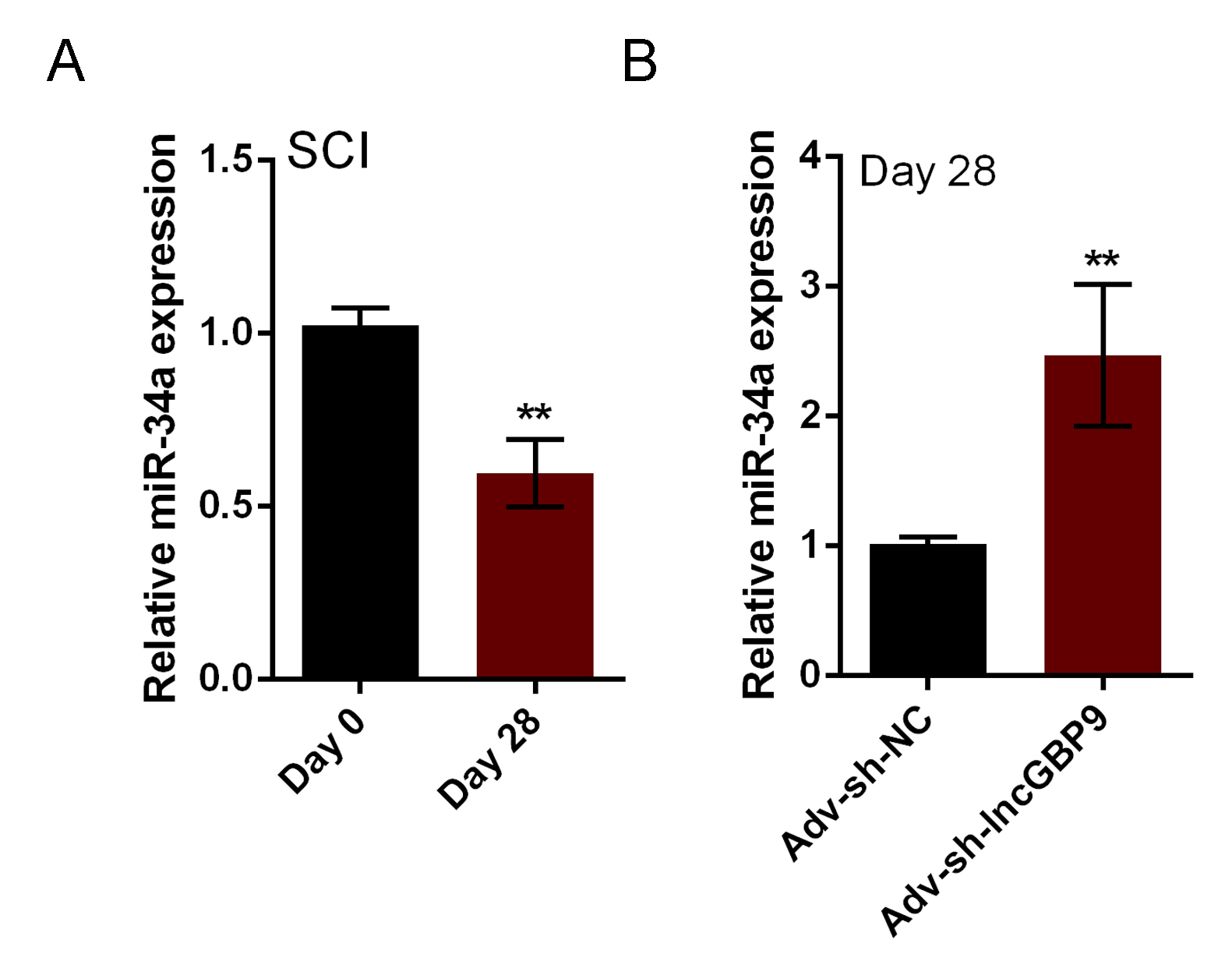

Supplement: Supplementary file 2 — Additional file 2: Figure S2. In vivo expression level of miR-34a in SCI mice (A) or Adv-sh-lncGBP9 infected SCI mice at day 28 of SCI treatment (B). Values are mean ± S.D of n = 5 independent experiments. [file 12974_2020_1805_MOESM2_ESM.tif]
