# Supplementary material for: LncGBP9/miR-34a axis drives macrophages toward a phenotype conducive for spinal cord injury repair via STAT1/STAT6 and SOCS3
Source: J Neuroinflammation. 2020 Apr 28;17:134. doi: 10.1186/s12974-020-01805-5 (PMC7187522; doi:10.1186/s12974-020-01805-5)
Supplement: Supplementary file 3 — Additional file 3: Table S1. The primer sequence [file 12974_2020_1805_MOESM3_ESM.docx]

Table S1 The primer sequence

| Name | Forward 5’-3’ | Reverse 5’-3’ |
| --- | --- | --- |
| QPCR-INOS | AGGGAATCTTGGAGCGAGTTG | CGTAATGTCCAGGAAGTAGGTGAG |
| QPCR-CD16 | AAGTGACACCCCATCCATCGTA | TCCTCCTTCCTCAGCCTCCTAA |
| QPCR-IFN-γ | ACTCAAGTGGCATAGATGTGGAA | ATGACGCTTATGTTGTTGCTGAT |
| QPCR-Arg1 | ATGAATGAGATTTATACGGTGCTGG | CTGCGGTGATAGGATGGGTGT |
| QPCR-CD206 | ACAGTCTGAGTGTACGCAGTGGT | AATGGGTTCAAGGTATTGTTTCC |
| QPCR-IL-4 | GAACGAGGTCACAGGAGAAGG | GATGAATCCAGGCATCGAAAA |
| QPCR-Arg1 |  |  |
| QPCR-lncGBP9 | TCTCAACATTCTCTGTTCCTGGG | CGCTCCTCTTCACCCCTTTCC |
| QPCR-lncFam91a1 | AAGGATTGTGGTGGAAGTTGGA | GGTGACCTGGACAGCCTAAGTG |
| QPCR-STAT1 | CCACGCCTTTGGGAAGTATTA | CATGGGAAGCAGGTTGTCTGT |
| QPCR-SOCS3 | AGAGCGGATTCTACTGGAGCG | CTGGATGCGTAGGTTCTTGGTC |
| QPCR-STAT6 | TCAAGATGACTGTGGAAAGGGAC | GAGTGGATGGACTGTGGAGGATA |
| QPCR-β-actin | TGCTGTCCCTGTATGCCTCTG | TTGATGTCACGCACGATTTCC |
| QPCR-miR-34a-5p | RT: GTCGTATCCAGTGCAGGGTCCGAGGTATTCGCACTGGATACGACACAACC  F:GCGGTGGCAGTGTCTTAGCT | R:CAGTGCAGGGTCCGAGGTAT |
| QPCR-U6 | CTCGCTTCGGCAGCACA | AACGCTTCACGAATTTGCGT |
| Adv-sh-NC | GGATCCTTCTCCGAACGTGTCACGTCTCGAGACGTGACACGTTCGGAGAAGAATTC | GAATTCTTCTCCGAACGTGTCACGTCTCGAGACGTGACACGTTCGGAGAAGGATCC |
| Adv-sh-lncGBP9 | GGATCCCCACAACAAATCCTTTCTTAACTCGAGTTAAGAAAGGATTTGTTGTGGGAATTC | GAATTCCCACAACAAATCCTTTCTTAACTCGAGTTAAGAAAGGATTTGTTGTGGGGATCC |
| Adv-lncGBP9 | AAACTGCAGGAGCAGAAGTGAGCCTGAGGAG | AAACTCGAGCCTCAAAGCAAACACCAGGATT |
| Si-NC | UUCUCCGAACGUGUCACGUTT | ACGUGACACGUUCGGAGAATT |
| Si-SOCS3 | UAGGAGACUCGCCUUAAAUTT | AUUUAAGGCGAGUCUCCUATT |
| SOCS3 overexpression | AAAGCTAGCCCATGGTCACCCACAGCAAGTT | AAACTCGAGTCCTTAAAGTGGAGCATCATAC |
| MiR-34a-5p mimics | UGGCAGUGUCUUAGCUGGUUGU | ACAACCAGCUAAGACACUGCCA |
| miR-34a-5p inhibitor | GAACAACCAGCUAAGACACUGC |  |
| NC mimics | UUCUCCGAACGUGUCACGUTT | ACGUGACACGUUCGGAGAATT |
| NC inhibitor | CAGUACUUUUGUGUAGUACAA |  |
| MiR-34a-5p mimics | UGGCAGUGUCUUAGCUGGUUGU | ACAACCAGCUAAGACACUGCCA |
| miR-34a-5p inhibitor | GAACAACCAGCUAAGACACUGC |  |
| **Luciferase** |  |  |
| Mmu-Wt-SOCS3-3’UTR | AAACTCGAGGAACTGCAAGGGGAATCTTCA | AAAGCGGCCGCTGCTACTGCCTCTTCACAACG |
| Hsa-Wt-SOCS3-3’UTR | AAACTCGAGGGGGAGTACCACCTGAGTCTC | AAAGCGGCCGCTGTGACCATTTCCTTCGCCAG |
| Mmu-Mut-SOCS3-3’UTR | AGAAATAACCACTCCCGCGCGCCAACCTAGGTGAGG | CCTCACCTAGGTTGGCGCGCGGGAGTGGTTATTTCT |
| Hsa-Mut-SOCS3-3’UTR | TGCTGAACTAATGAGAAGCGCGAGGGAATCTTCAAACTT | AAGTTTGAAGATTCCCTCGCGCTTCTCATTAGTTCAGCA |
| Wt-GBP1P1 | AAACTCGAGTCAGGTGTCTAATTGCATCAC | AAAGCGGCCGCTTGGAAACTTTGTTTCTTCTC |
| Wt-GBP9 | AAACTCGAGCATGGCTGTCTTCATGGAGCAT | AAAGCGGCCGCTGTGACTGGAGAAAGTTTTGGA |
| mut-GBP1P1 | AGCCTGAACTGGCTAGCGGCAAGTCTGGTCACTAAAC | GTTTAGTGACCAGACTTGCCGCTAGCCAGTTCAGGCT |
| mut-GBP9 | GAGGCATCGAATAAATAGCGGCAGGAAGAACTGGATCG | CGATCCAGTTCTTCCTGCCGCTATTTATTCGATGCCTC |
| wt-miR-34a promoter LUC | AAAAGATCTTACCCCCTTTCTAAGACAAAATCC | AAAGCTAGCGCCCGCAGTCACAGGAAGAT |
| mut1- miR-34a promoter LUC | GTGTGGCAAATACTGTTCGCGCGCGCGACATTTGCAGCCTC | GAGGCTGCAAATGTCGCGCGCGCGAACAGTATTTGCCACAC |
| STAT6 overexpression | AAAGCTAGCCATGTCTCTGTGGGGCCTAATT | AAACTCGAGTTCTCCAGCTGGGATCACCAGC |
| Si-STAT6 | CCGGGAUCUUGCUCAGUUATT | UAACUGAGCAAGAUCCCGGTT |
| Chip-QPCR-site1 | TGTCGTGTGTGGCAAATACTGTT | TCTATCATACTCCTCCGGGGCTC |
| Chip-QPCR-site2 | CCAGCCCAGTCTCTGCATATTA | TGGAGTCCTTCACACCAACACC |
